# Nanopore sequencing combined with adaptive sampling and NanoExpansion enables accurate characterization of repeat expansion disorders

**DOI:** 10.1093/nargab/lqag117

**Published:** 2026-10-06

**Authors:** Luca Morandi, Francesco Casadei, Sara De Fanti, Luigi Bonan, Francesco Ravaioli, Flavia Palombo, Alessia Fiorentino, Silvia de Pasqua, Veria Vacchiano, Patrizia Avoni, Giovanni Rizzo, Sabina Capellari, Edoardo Ruggeri, Vincenzo Donadio, Gastone Castellani, Maria Giulia Bacalini, Raffaele Lodi, Rocco Liguori, Valerio Carelli, Caterina Tonon

**Affiliations:** IRCCS Istituto delle Scienze Neurologiche di Bologna, Bologna, 40139, Italy; Functional and Molecular Neuroimaging Unit, Bellaria Hospital, Department of Biomedical and Neuromotor Sciences, University of Bologna, Bologna, 40126, Italy; IRCCS Istituto delle Scienze Neurologiche di Bologna, Bologna, 40139, Italy; IRCCS Istituto delle Scienze Neurologiche di Bologna, Bologna, 40139, Italy; IRCCS Istituto delle Scienze Neurologiche di Bologna, Bologna, 40139, Italy; IRCCS Istituto delle Scienze Neurologiche di Bologna, Bologna, 40139, Italy; IRCCS Istituto delle Scienze Neurologiche di Bologna, Programma di Neurogenetica, Bologna, 40139, Italy; IRCCS Istituto delle Scienze Neurologiche di Bologna, Programma di Neurogenetica, Bologna, 40139, Italy; IRCCS Istituto delle Scienze Neurologiche di Bologna, Bologna, 40139, Italy; IRCCS Istituto delle Scienze Neurologiche di Bologna, Bologna, 40139, Italy; IRCCS Istituto delle Scienze Neurologiche di Bologna, Bologna, 40139, Italy; Department of Biomedical and Neuromotor Sciences, University of Bologna, Bologna, 40126, Italy; IRCCS Istituto delle Scienze Neurologiche di Bologna, Bologna, 40139, Italy; IRCCS Istituto delle Scienze Neurologiche di Bologna, Bologna, 40139, Italy; Department of Biomedical and Neuromotor Sciences, University of Bologna, Bologna, 40126, Italy; IRCCS Istituto delle Scienze Neurologiche di Bologna, Bologna, 40139, Italy; IRCCS Istituto delle Scienze Neurologiche di Bologna, Bologna, 40139, Italy; Department of Biomedical and Neuromotor Sciences, University of Bologna, Bologna, 40126, Italy; Department of Medical and Surgical Sciences, University of Bologna, Bologna, 40126, Italy; IRCCS Azienda Ospedaliero - Universitaria di Bologna, Bologna, 40138, Italy; IRCCS Istituto delle Scienze Neurologiche di Bologna, Bologna, 40139, Italy; Department of Biomedical and Neuromotor Sciences, University of Bologna, Bologna, 40126, Italy; IRCCS Istituto delle Scienze Neurologiche di Bologna, Bologna, 40139, Italy; Department of Biomedical and Neuromotor Sciences, University of Bologna, Bologna, 40126, Italy; IRCCS Istituto delle Scienze Neurologiche di Bologna, Bologna, 40139, Italy; Department of Biomedical and Neuromotor Sciences, University of Bologna, Bologna, 40126, Italy; IRCCS Istituto delle Scienze Neurologiche di Bologna, Programma di Neurogenetica, Bologna, 40139, Italy; Department of Biomedical and Neuromotor Sciences, University of Bologna, Bologna, 40126, Italy; IRCCS Istituto delle Scienze Neurologiche di Bologna, Bologna, 40139, Italy; Functional and Molecular Neuroimaging Unit, Bellaria Hospital, Department of Biomedical and Neuromotor Sciences, University of Bologna, Bologna, 40126, Italy

## Abstract

Expansion repeat disorders are caused by abnormal expansions of short tandem repeats (STRs). We evaluated targeted long-read nanopore sequencing for comprehensive characterization of STR expansions in 132 participants, including patients with myotonic dystrophy type 1 (DM1, *n* = 51) and DM2 (*n* = 3) already characterized by Southern blot, *C9orf72*-associated amyotrophic lateral sclerosis (ALS *n* = 11) evaluated by repeat primed polymerase chain reaction (RP-PCR; validation group), suspected spinocerebellar ataxia (SCA, *n* = 46), cerebellar ataxia with neuropathy and vestibular areflexia syndrome (CANVAS; *n* = 13), Huntington’s disease (1), Kennedy disease (1), Friedreich ataxia (FRDA, 2), and 4 healthy controls (test group). Native DNA was sequenced using adaptive sampling (AS) on Oxford Nanopore GridION or PromethION-2Solo platforms. Long-PCR sequencing was performed for comparison. We also developed NanoExpansion, a Python-based tool for sizing expanded alleles and detecting sequence interruptions. We correctly characterized all DM1, DM2, ALS, FRDA, Huntington’s disease, and Kennedy disease cases, and identified eight SCA27B, two SCA37, three SCA31, one SCA3, and nine CANVAS cases. Compared with the AS approach, long-PCR underestimated alleles exceeding 800 repeats. Repeat interruptions were detected in 27/51 (52.9%) DM1 cases and 1/10 (10%) ALS, but not in CANVAS, FRDA, Huntington’s disease, Kennedy disease, or SCA cases. This integrated sequencing analysis pipeline provides accurate, comprehensive characterization of repeat expansion disorders and has the potential to improve routine genetic diagnosis.

## Introduction

Short tandem repeats (STRs), also known as microsatellites, are repeating tracts of DNA, usually 2–12 bp long, that can be found in both coding and noncoding regions of the genome [1]. They are characterized by high length polymorphism and instability which results in >50 different diseases [2], that are called repeat expansion disorders (REDs) [3], as listed in STRchive (https://strchive.org) [4].

Among those, myotonic dystrophy type 1 (DM1) is the most common muscular dystrophy in adults, with an estimated prevalence of about 1/8000 worldwide [5]. It is an autosomal dominant disorder and can involve multiple organs and systems [6]. DM1 is caused by the expansion of the unstable CTG trinucleotide repeat in the 3′ untranslated region of the *DMPK* gene, located on chromosome 19q13.32 [7]. The size of the expansion can greatly vary with healthy people showing a range between 5 and 35 CTG repeats. Patients with a CTG expansion from 36 and 49 are asymptomatic, and do not meet diagnostic criteria for DM1. However, these alleles are unstable during transmission, particularly through meiosis, and can expand into the pathogenic range in the next generation (premutation or mutable normal alleles). Affected patients have a CTG expansion >50. Expansion size is associated with age of onset and severity: mild late-onset, classic and congenital DM1 are associated with expansions ≤150 CTGs (E1), ≤1000 CTGs (E2), and ≥1000 CTGs (E3), respectively [8]. Clinical features in DM1 patients vary according to severity group. Most common symptoms are distal muscle weakness and myotonia [7]. However, the high individual variability in DM1 patients of the same subtype makes the genotype–phenotype correlation not completely understood. The great instability of CTG expansion in both somatic and germline cells, which persists during patient’s lifetime, is called somatic mosaicism phenomenon [9]. Another crucial aspect that makes the genotype–phenotype correlation more complex is the presence of interruptions within the CTG expansion. Several variant expansions, such as CCG, CGG, CTC, TTG, GGC, CAG, have been observed, especially at the 3′ and 5′ ends of the *DMPK* gene [10, 11]. These interruption motifs have a stabilizing effect of the expansion on germline transmission. However, the great heterogeneity in terms of motif, length and location of the interruption patterns along the CTG expansion leads to a huge phenotypic variability, which deserves to be further investigated [12].

Other REDs are spinocerebellar ataxias (SCAs), which are a heterogeneous group of neurodegenerative autosomal dominant diseases (prevalence of about 3/100 000 [13, 14]. Despite their complex genotype–phenotype spectrum, common clinical features among SCAs are progressive cerebellar ataxia, with oculomotor abnormalities, visual problems, pyramidal and extrapyramidal signs, dysarthria and cognitive dysfunction [13, 15].

Cerebellar ataxia with neuropathy and vestibular areflexia syndrome (CANVAS) has recently been included among the REDs [16]. It is an autosomal recessive neurodegenerative balance disorder, caused by biallelic repeat expansion mutations in the *RFC1* gene, characterized by the peculiar clinical phenotype including cerebellar ataxia, sensory neuronopathy (ganglionopathy), bilateral vestibular hypofunction, and chronic cough. Huntington’s disease and Kennedy disease are two other well-known REDs.

The current diagnosis of REDs is mainly based on two well-known and standardized techniques: Southern blot (SB) assay and triplet-repeat primed polymerase chain reaction (RP-PCR), often coupled. SB is considered the gold-standard, but it is labor-intensive and time-consuming, it requires large quantities (≥10 µg) of high-quality DNA to produce valuable results [17], it is inaccurate in repeat expansion estimates, and suffers from low resolution and sensitivity in detecting short expansions. SB also lacks in providing a sequence context on the evaluated STRs [18].

RP-PCR is relatively inexpensive, easy to implement and more efficient in detecting short expansions with respect to SB. However, it cannot reveal long repeated regions and mosaicisms. Furthermore, it cannot determine the size of the expansion and is prone to errors due to amplification artifacts [19]. The high concentration of GC in some repeat and interruption motifs causes the allelic drop out polymerase chain reaction (PCR) bias, leading to further sequencing errors.

The recent third generation long-read sequencing (LRS) technologies, such as Oxford Nanopore Technologies (ONT, Oxford Science Park, UK) or PacBio (Menlo Park, CA), allow to obtain long DNA fragments and to accurately characterize the size of expansions, mosaicisms and interruption patterns [20–22]. Long reads enable robust structural variation and tandem repeats detection. In addition, nanopore sequencing provides an in-silico target sequencing technique, named adaptive sampling (AS) [23]. During a sequencing experiment, if the basecalled sequence does not fall within the region of interest provided to the machine beforehand, the sequencing process stops and the DNA fragment is ejected from the pore. A new fragment then begins to be sequenced. This application avoids using PCR for target sequencing, that can introduce artefacts, biases and may generate false negative results [23–25].

Several computational tools, including Straglr [26], LongTR [27], STRdust [28], and STRkit [29], have been developed to estimate repeat lengths and genotype expanded alleles from LRS data. Although these tools provide reliable repeat sizing and, in some cases, allele phasing or motif annotation, their primary objective remains repeat genotyping. However, the repeat pattern architecture of expanded regions, such as motif interruptions, internal sequence organization, and DNA methylation, can significantly influence disease characterization and clinical severity. Therefore, to address the need of a more comprehensive analysis of repeated regions, we developed NanoExpansion, a prototype software designed to reconstruct and characterize the complete architecture of expanded alleles from long read sequencing data.

In addition to repeat sizing, NanoExpansion identifies motif interruptions, performs allele clustering, and extracts locus-specific DNA methylation profiles directly from the sequencing reads, providing an integrated genetic and epigenetic characterization of repeat expansion loci. Moreover, NanoExpansion also provide a complete and detailed report to guide and support medical doctors in the diagnosis of patients with REDs. This comprehensive framework enables a more detailed investigation of genotype–phenotype relationships than currently available repeat genotyping software.

This study aimed to demonstrate the potential diagnostic power of nanopore sequencing by characterizing repeat expansion regions in two cohorts. The first cohort comprised patients with DM1, DM2, or amyotrophic lateral sclerosis (ALS) carrying a *C9orf72* expansion for whom a genetic diagnosis had already been made. The second cohort comprised subjects with suspected SCA, CANVAS, HD, or Kennedy disease. A comprehensive bioinformatics pipeline has been developed. The present work would reinforce the findings of previous research studies using the latest human genome reference (hs1), which was provided by the Telomere-to-Telomere (T2T) Consortium [23, 24, 30, 31].

## Materials and methods

### Patient consent

The study has been approved by the AUSL Bologna Ethical Committee (BITRO N° 599–2022-SPER-AUSLBO). This study was conducted in agreement with either the most updated Declaration of Helsinki and all the international and local laws applied to clinical studies and patient protection.

### Study setting and data collection

The dataset included 132 participants enrolled at the IRCCS Istituto delle Scienze Neurologiche di Bologna, Bellaria Hospital, Italy. The cohort included 51 individuals with genetically confirmed diagnoses of DM1, three DM2, 11 ALS cases, of which 10 already identified for expansion in *C9orf72* with RP-PCR (validation group). Additionally, the cohort comprises patients with clinical diagnoses (suspected cases, test group) of SCA (46), CANVAS (13), Friedreich ataxia (FRDA, two), Huntington’s disease (HD, one), and Kennedy disorder (one). Four healthy controls (mean age: 71) were also included.

### DNA purification

DNA was purified from whole blood samples, using the Maxwell^®^ 16 Blood DNA Purification Kit (Promega, Madison, WI 53711 USA). DNA concentration was measured using Quantus Fluorometer (Promega, Madison, WI 53711 USA).

### Library preparation for ONT platform

Two different types of libraries were processed: one from native DNA and one for long PCR product. For native DNA, the Ligation Sequencing Kit V14 (SQK-LSK114, Oxford Nanopore, Oxford Science Park, UK) was used starting with 2 μg of total DNA following the protocol of the provider. In brief, DNA was incubated with DNA-repair and end-repair enzymes in thermal cycler at 20°C for 10 min and 65°C for 5 min. It followed AMPure XP bead purification (Beckman Coulter, Indianapolis, IN) and 20 min incubation with Quick T4 DNA Ligase (NEB, Ipswich, MA) and the Ligation Adapter. After AMPure XP purification with Long Fragment Buffer (LFB), the concentration was measured by Quantus™ Fluorometer (Promega, Madison, WI 53711 USA), Fragment Analyzer 5200 (Agilent, Santa Clara, CA) and NEB Bio Calculator (New England Biolabs, Ipswich, MA, USA) or Promega Biocalculator (Promega, Madison, WI 53711 USA). Then, 200–800 ng of DNA were loaded onto the R10.4.1 flow cell (FLO-MIN114) using the ONT GridION platform (ONT, Oxford UK). Twenty-one cases were loaded onto PromethION 2 Solo (ONT, Oxford UK) to increase the coverage depth. To reduce the costs, the P2Solo setup was optimized by reusing the same flow cell with a running time of 16 h. After DNase treatment, libraries were loaded with no barcoding step to maximise the coverage depth.

For long PCR product, the Native Barcoding Kit 24 V14 (SQK- NBD114.24) was used including 24 different samples. Primers used for all loci and PCR conditions are described in Supplementary File 1. In brief, 400 ng of purified amplicon pools were treated with NEBNext FFPE DNA repair and Ultra II End-Prep enzyme mix (NEB, Ipswich, MA), at 20°C and at 65°C for 10 min each, purified with AMPure XP beads and barcoded with NEB Blunt/TA Ligase Master Mix (NEB, Ipswich, MA), for 20 min at room temperature. After pooling and repurification with AMPure XP beads, DNA was ligated with Native Adapter (NA) using Quick T4 DNA ligase (NEB, Ipswich, MA) for 20 min at room temperature. After the final bead purification using LFB to enrich DNA >3 kb, the library was evaluated with Quantus™ Fluorometer (Promega, Madison, WI 53711 USA). Depending on the fragment size, 200–800 ng of DNA were loaded onto washed and reused R10.4.1 flow cell (FLO-MIN114) coming from AS experiments using the ONT GridION platform (ONT, Oxford UK).

### Native nanopore sequencing downstream analysis

During nanopore sequencing, the MinKnow software (version 6.2.14, ONT) stored the raw current signal into POD5 files. Real-time basecalling was set up to HAC algorithm to avoid overloading the GridION computing unit. MinKnow also performed AS enriching for 37 loci of interests (see Supplementary Tables S1 and S2). Those loci involved classical expansion repeat regions, as well as a list of 13 genes in which (CAG)_>5_ polyQ repeats were detected using blat tool (https://genome.ucsc.edu/cgi-bin/hgBlat) in the human genome. The latter have not yet been associated with any disease. Each sequencing run usually lasted 16–24 h. New MinION flow cells were used for native DNA processing, while used and washed flow cells were used for long amplicons. After sequencing, the raw signal was transferred to a high-performance computing station (equipped with a single AMD EPYC 9654 processor (96 cores, 192 threads, base clock 1.5 GHz, boost up to 3.7 GHz) and two NVIDIA L40 GPUs (45 GiB memory each, CUDA 12.2); the system ran Debian GNU/Linux 6.1.0–29-amd64) and basecalled with Dorado, version 1.3.0, models v5.2.0. Samples were basecalled with both high accurate (hac, a part of them) and super accurate (sup) Dorado models. The q score filter was set to 10. The basecalled sequences were aligned to the reference genome (GRCh38 and T2T CHM13v2/hs1) using minimap2 (version 2.26) [32]. All native samples were aligned to the GRCh38 reference (hg38), and seven subjects with DM1 were also mapped to hs1 (T2T). Samples sequenced with long PCR were aligned to T2T CHM13v2/hs1. Aligned reads were stored in BAM files and indexed. The aligned data were visually inspected with IGV [33]. Single nucleotide variants were called using Clair3 (version 1.0.10) [34]. SNP information was used to perform haplotyping with Whatshap (version 2.4) [35]. Structural variants were called with Sniffles (version 2.2) [36], CuteSV (version 2.1.0) [37, 38], while STRs were detected using several tools, such as Sniffles (version 2.2), CuteSV(version 2.1.0), Nanorepeat [39], Straglr (version 1.4.1)(26), STRdust (version 0.20.0) [28], STRkit (version 0.24.2) [29], and LongTR (version 638942f) [27]. The VCF output files were annotated with Stranger (version 0.9.5, doi:10.5281/zenodo.4548873). Finally, NanoExpansion, which is a prototype software developed within our research group, was used to characterize repeat expansion patterns, detecting the main motifs and the interruptions. The whole pipeline was integrated into a Snakemake [40] workflow.

### NanoExpansion

NanoExpansion is a Python-based software package designed to reconstruct the internal architecture of pathogenic repeat expansions from LRS data. By providing an aligned BAM- file and the genomic coordinates of a target repeat locus, the software extracts spanning reads and iteratively identifies repeat and interruption motifs within each expanded allele. Unlike repeat genotyping tools that primarily estimate repeat expansion size, NanoExpansion reconstructs the sequential pattern of repeat blocks, filtering spurious nucleotides, and generated a compact representation of the expanded allele [e.g. (CTG)n(CCG)m(CTG)k]. In addition, NanoExpansion computes allele-specific summary statistics, extracts DNA methylation profiles across the target locus, and produces a detailed report to support medical doctors in the diagnostic decision process. Users provide genomic coordinates of the target locus, together with canonical repeat motif and a group of interruption motifs to be investigated (if not specified, NanoExpansion can also automatically detect interruption motifs), allowing the tool to characterize a broad range of REDs. Additional filtering parameters could be provided by the user. NanoExpansion is designed to work with hg38 and T2T genome references. The software has been benchmarked on a set of artificially expanded reads (see Supplementary Material) to prove its accuracy in detecting expansion patterns.

### Library preparation for short read exome sequencing

To rule out possible false calls coming from ONT, we performed a separate test on five samples already evaluated with exome sequencing using NovaSEQ6000 for all loci listed in Supplementary Table S1. The sample library for exome sequencing was prepared using xGen™ DNA EZ Library Prep Kit (IDT, Coralville, Iowa, USA), enriched with xGen Exome Research Panel v2 probes, and then sequenced with 2 × 150 bp paired end reads on a NovaSeq 6000 instrument (Illumina). Bioinformatic analysis followed the GATK v.4.2.0.0 workflow for germline variant discovery, aligning to reference genome GRCh38/hg38. BAM files were visualized on IGV.

### Triplet-RP-PCR for C9orf72

*C9orf72* repeat was typed using the AmplideXPCR/CE *C9orf72* Kit (Asuragen, Austin Texas, USA) amplifying the target using a three-primer G4C2-RP-PCR configuration [41] followed by fragment sizing on a capillary electrophoresis instrument. The sizing of amplicons was performed by GeneMapper software according to the manufacturer’s protocols.

### SB for *DMPK* expansion repeats

SB data analysis was performed as previously described [42].

### Statistical analysis

The comparison between using Dorado hac and sup models have been assessed by computing the following metrics: mismatch, insertion, deletion, and homopolymer error rate (homopolymers ≥ 4 bp, that is known to be an ONT-specific bias). Statistically significant differences have been verified using the Wilcoxon test (α = 0.05, one-tailed). Agreement at read level (i.e. comparing both BAM files) was assessed by computing mean and 95% confidence interval of the difference inedit distance (ΔNM, global measure of error per read), mapping quality (ΔMAPQ, which measures mapping efficiency), and in the number of clipped bases, that indicates partial or problematic alignments. Same statistical procedure has been used for comparing alignment to hg38 and T2T/hs1 reference genome.

Effectiveness of AS was measured by computing enrichment ratio (ER) and fold-enrichment (FE, using a control samples, sequenced both with and without AS); mean ER and FE have been provided, together with 95% confidence interval (ER ≥ 5×, FE ≥ 4× can be considered good efficacy). All these metrics have been computed using Python 3.6.13 (Pysam 0.21.0 and Pybedtools 0.9.0 packages).

The agreement between native ONT and SB has been measured considering that SB only provided intervals of expansion size. First, considering only genomic classification (E1, E2, and E3), ordinal metrics such as quadratic weighted Cohen’s Kappa (${{\kappa }_w}$), ordinal accuracy, and mean absolute error (MAE) have been used. These metrics evaluate ordinal classification, considering that a misclassification E1–E2 or E2–E3, is less severe than E1–E3. Genomic classes have been assigned to native nanopore sequencing according to the mean of the distribution of repeat sizes. In addition, metrics evaluating the overlapping between nanopore and SB results have been used: coverage rate of native ONT within the SB intervals, distance of the median of native ONT from SB intervals, and probabilistic overlap between a uniform distribution defined by SB intervals and a kernel density estimate of native ONT.

Native and long PCR ONT were compared by drawing scatter plots, fitting a linear model (R^2^ is reported), and computing both the Pearson correlation coefficient (ρ) and the Lin’s Concordance Correlation Coefficient (CCC, that measures the degree to which observations are close to the 45° line) by disease. Native ONT results have been obtained from IGV, while for long PCR, most frequent reads covering both repeated loci and flanking regions were considered. Comparison between long PCR and native ONT has been conducted using R 4.3.1.

Metrics used to compare the different variant caller software tools against the IGV ground truth spanned accuracy, completeness, and agreement measures. Accuracy and error dispersion were quantified using MAE, root mean squared error (RMSE), bias, standard deviation of errors (Std Error), interquartile range error (IQR), and coefficient of variation of errors. Completeness was assessed via the hit rate (HR) relative to ground truth (number of calls over the calls of the ground truth). To penalize tools with low call rates, HR-adjusted metrics were computed, including weighted MAE, weighted RMSE, and a combined score.

To estimate reliability and concordance between tools and ground truth, Intraclass Correlation Coefficient, and CCC have been used, respectively. To estimate parameter uncertainty, 95% confidence intervals were derived using nonparametric bootstrap resampling (2000 iterations). In addition, Bland–Altman plots have been drawn for visual evaluation. All these metrics have been computed using Python 3.6.13. All the listed metrics are mathematically defined in the Supplementary Material.

## Results

A summary of nanopore sequencing quality by comparing basecalling models, reference genomes, coverage, enrichment, and motif detection is reported in Fig. 1. Samples were basecalled using both hac (version 5.0.0) and sup (version 5.0.0) Dorado models. These two models differ significantly in terms of time to return the basecalled reads (sup model takes at least 2–3 additional hours with respect to hac model) and of quality of basecalled reads. Distributions of the computed metrics for hac and sup models are shown in Fig. 1A and listed in Supplementary Table S3. Basecalling with super accurate Dorado model clearly reduced the total amount of error (Wilcoxon test, *P* <.05 for each metric). Mean values and 95% confidence intervals of ΔNM, ΔMAPQ, and difference in number of clipped bases are −6.305 [−6.322, −6.289], 0.626 [0.623, 0.629], and −58.42 [−58.53, −58.31], respectively, confirming that sup model is more reliable and stable. Similarly, mapping reads to T2T reference resulted in a general decreasing of mismatch, insertion, deletion, and homopolymer error rates (*P* <.05 for all metrics, Wilcoxon test). Violin plots are shown in Fig. 1B (see also Supplementary Table S4).

**Figure 1.**
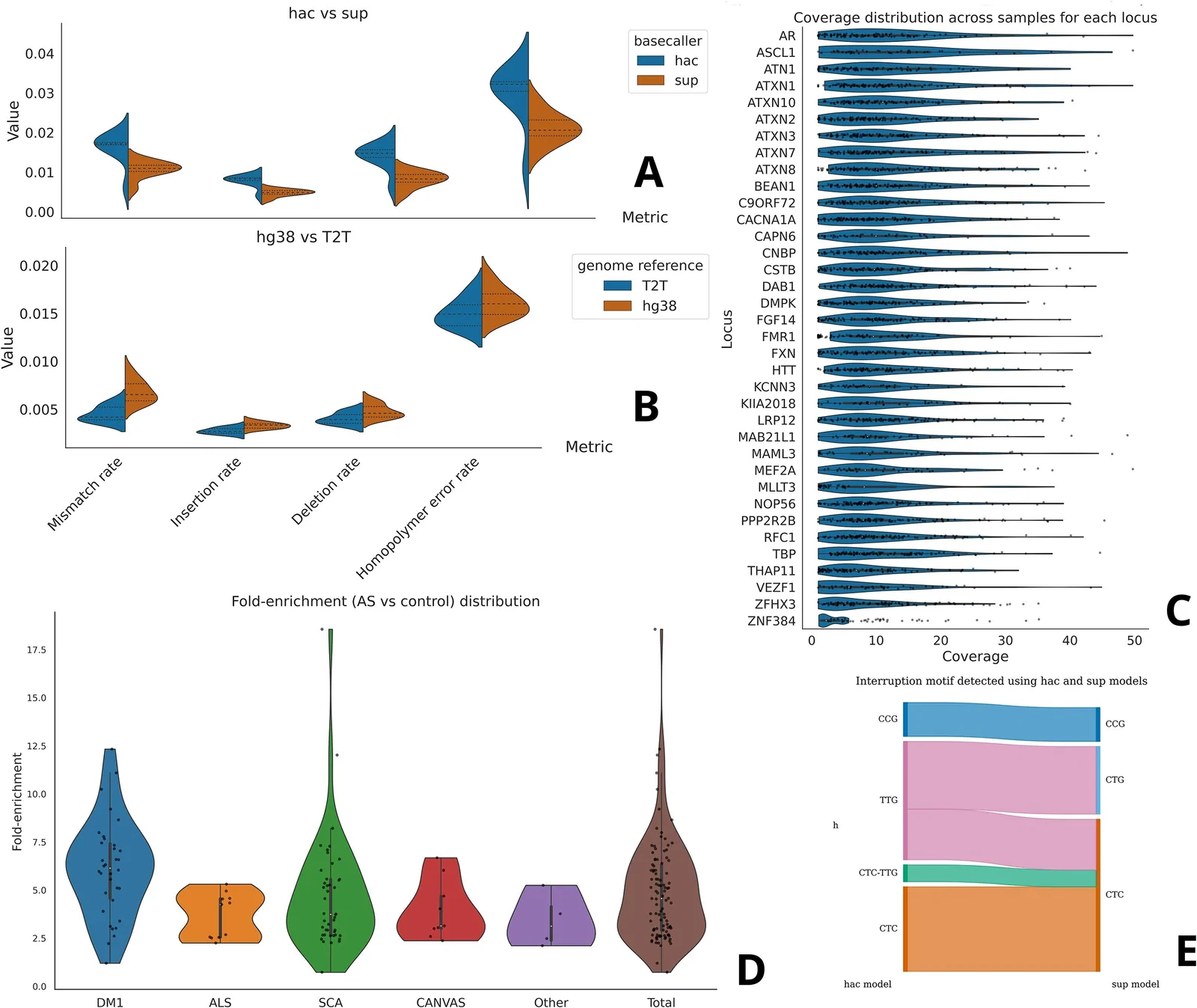
Quality assessment of nanopore sequencing. (**A**) Violin plots of the metrics (mismatch, insertion, deletion, and homopolymer error rate) computed for samples basecalled with both hac and sup Dorado models (hg38 genome reference) and (**B**) aligned to T2T and hg38 reference genome (Dorado model, version 1.3.0, sup v5.2.0). The use of super accurate basecaller (orange) clearly reduced the total amount of errors (*P* <.05 for each metric, Wilcoxon test) with respect to hac basecaller (blue). Similarly, samples aligned to T2T reference (blue) showed a smaller number of errors (*P* <.05 for each metric, Wilcoxon test) with respect to mapping to hg38 (orange). (**C**) Violin plots of the coverage of each locus targeted by the AS. The median coverage on target loci is 10×. However, it clearly ranges from relatively low values (bottom) to higher values, reaching up to 20–30× for some samples. (**D**) Violin plots of the distribution of the FE (AS versus control) for each disease group and considering all samples. The use of AS clearly enriched for the targets of interest. Samples diagnosed with CANVAS and ALS appear to be less enriched. (**E**) Sankey plot showing how the detected interruption motifs change between hac (left) and sup (right) basecalling. TTG pattern always disappears, while CCG motif always survives. Even if the sup model may not represent the ground truth given the known challenges with STRs, it could be considered more reliable, as it calls more biologically plausible interruption motifs. It could be helpful in determining which kind of errors were made by the high-accuracy model.

An example of a DM1 case comparing hac and sup model is shown in Supplementary Fig. S1. The IGV display shows more point mutations in the hac model (bottom), which is due to the lower accuracy with respect to the sup model (top). Moreover, the sizes of STRs are slightly different (hac model usually tends to overestimate the inserted repeats).

The regions of interest had a median coverage of 10×, indicating that AS enrichment worked effectively. Distributions of the coverage for each target region are shown in Fig. 1C (see also Supplementary Table S5). Relatively low coverage was associated with a lower quantity of DNA available for preparing the library (i.e <2 μg). The AS effectiveness resulted in an ER of 5.79× [5.12, 6.47] and in an FE of 5.24× [4.63, 5.85] (mean and 95% confidence interval). The distribution of FE according to each disease is shown in Fig. 1D, while ER distribution is reported in Supplementary Fig. S2 (values are listed in Supplementary Table S6).

Unlike SB, one of the main advantages of nanopore sequencing is to fully characterize the repeat expansion pattern in *DMPK* and in other genes of interest, revealing also interruption motifs. Since several DM1 samples were basecalled using both hac and sup models, we compared the interruption motifs detected in both results. The Sankey plot in Fig. 1E illustrates the changes in interruption motifs in the same samples between hac and sup basecalling (hac motifs on the left, sup on the right).

After super accurate basecalling, only CTC motif in 17 cases and CCG motif in two cases survived. Long PCR products were also inspected for interruptions, finding CTC interruptions in 15 cases, and confirming the CCG interruptions in two individuals.

Figure 2A shows the CTG repeat sizes in the *DMPK* gene estimated using native long reads, for 34 samples affected by DM1, alongside the SB evaluation. The boxplots refer to native ONT results, obtained from visual inspections in IGV, while SB intervals are represented as rectangles. All patients with DM1 were confirmed by ONT sequencing having >50 repeats. Both patients with short and long expansions were correctly detected (Fig. 2C, case dm_18 and dm_17 respectively).

**Figure 2.**
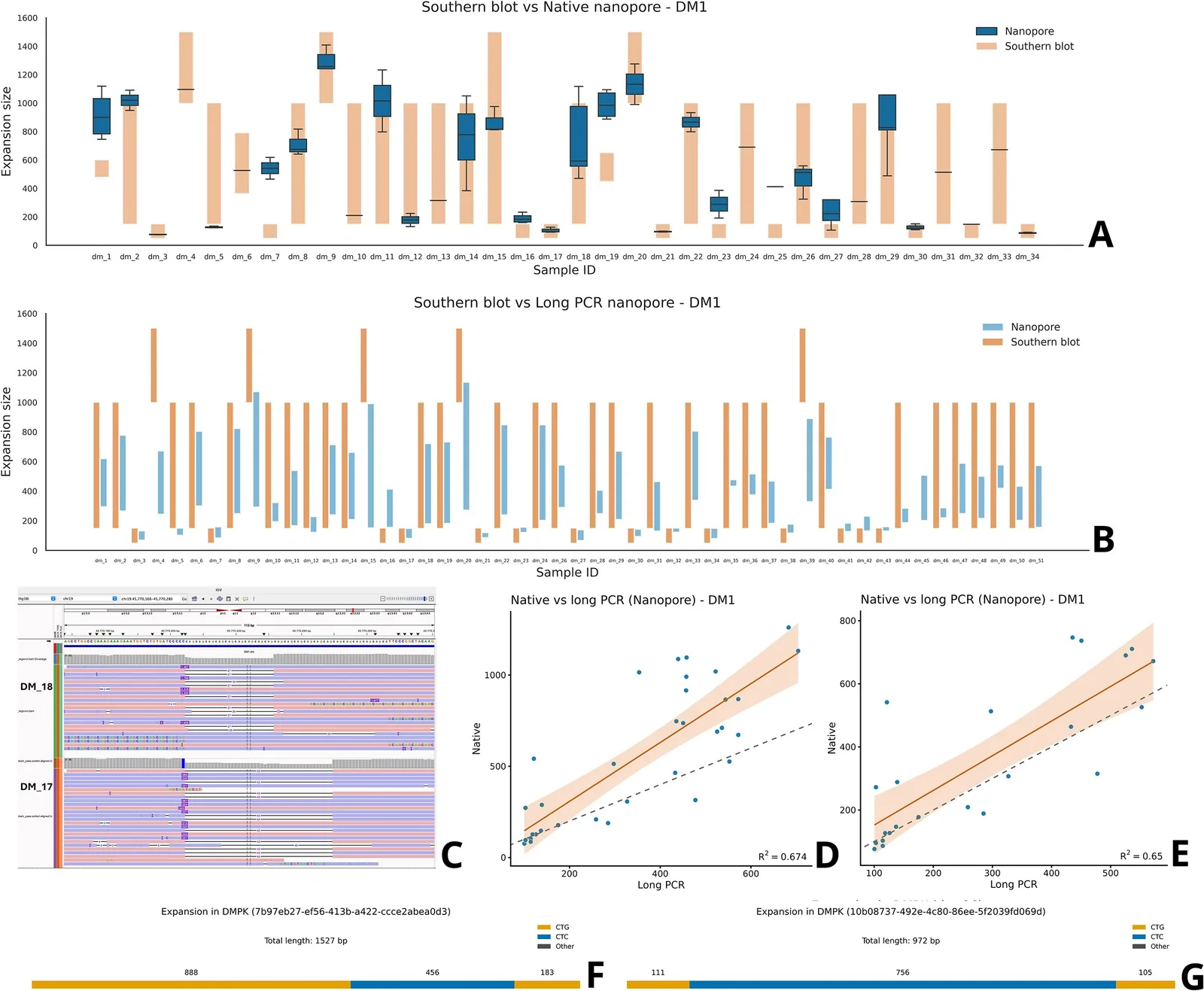
Analysis of estimates of repeat size for DM1 samples. (**A**) Boxplots of the nanopore sequencing STR estimates and SB quantification (rectangles) for DM1 patients. Apart from six cases, nanopore sequencing agrees with SB and provides a more accurate estimate of the STR size. Samples with wider boxplots were characterized by the mosaicism phenomenon. (**B**) Distribution of the estimates of expansion size for DM1 patients obtained with long PCR nanopore (blue rectangles) and SB quantification (orange rectangles). Long PCR allowed to obtain more precise estimates, and it agreed with SB classification. As mentioned, long PCR failed when the expansion size is relatively large (e.g. case dm_39). (**C**) Graphical visualization using IGV showing two cases of DM1 expansion: dm_18 revealed a large expansion until 1131 repeats; dm_17 was detected to have until 135 repeats. This clearly demonstrates that ONT is suitable for the detection of both short and long repeat expansions. (**D**) Scatter plot and linear model for native versus long PCR nanopore estimates of expansions for DM1 samples. The 45° line is reported (dashed). The Pearson correlation coefficient is 0.821, while CCC is 0.540. (**E**) After removing estimates larger than 800 repeats, ρ becomes 0.806, while CCC increases up to 0.728. In general, the agreement between native and long PCR can be considered good enough to use long PCR as an orthogonal method for native ONT in DM1, especially for relatively short repeats. (**F, G**) NanoExpansion plots of two mutated reads read for a DM1 patient in gene *DMPK*. The main expansion motif, CTG, is colored in orange, the main interruption motif in blue, while other secondary interruptions in dark gray. The number above the plot indicates the total length of the portions. NanoExpansion provides a simple and immediate tool for investigating at repeat expansion patterns in REDs..

A total of 28 out of 34 subjects (82%) with DM1 agreed with SB data. The κ_w_ with quadratic weights is 0.68 [0.41, 0.86], while the MAE is 0.24 [0.12, 0.38]. Considering the overlap between the distribution of native ONT estimates and SB intervals, the median of coverage ratio is 1 (with 53%, 11%, 18%, and 18% of cases falling within [0.75, 1], [0.5, 0.75], [0.25, 0.5], and [0, 0.25], respectively), while for distance to confidence interval is 0 (in 76% of cases, distance is equal to 0, while the median distance for remaining ones is 51.75; IQR = 0), and overlap is 0.41 (0.22 and 0.55 for 25% and 75% quantile, respectively).

Long PCR and SB data comparison in 50 samples are shown in Fig. 2B, both represented as rectangles. Nanopore sequencing with long PCR provided thousands of reads, due to PCR duplicates, varying in size (Supplementary Fig. S3). Only reads spanning the repeat and the flanking sequences until the primers were selected for analysis and used to plot rectangles. In this regard, the SB class was confirmed in 41 out of 50 samples (82%). Long PCR validated native DNA estimates in DM1 samples for relatively small repeat expansions (≤800 repeats), while this approach tends to underestimate larger expanded alleles in five cases. Fitting a linear model, the Pearson correlation coefficient between native and long PCR is 0.821, while CCC is 0.540, Fig. 2D. After removing estimates >800 repeats, ρ became 0.806, while the CCC increased up to 0.728 (Fig. 2E).

In Supplementary Fig. S4, dm_1 case is shown comparing native DNA and long PCR analysis. In Supplementary Table S7, a comparison of the number of repeats found using SB, native DNA and long PCR analysis is shown, where the entire repeat patterns characterized with NanoExpansion are reported for patients affected by DM1. An example of the plots produced by NanoExpansion for an expanded alleles of a DM1 patient (dm_26) is shown in Fig. 2F and G. Main expanded repeat motifs are colored in orange, main interruption motifs (e.g. CCG, CTC, the most biologically plausible ones) are reported in blue, while secondary interruptions (alternative variants, that might also be due to basecalling errors) are shown in dark gray. Interruption patterns were found in 27/51 (52.9%) of the DM1 cases. Long PCR confirmed these findings with regard to native DNA analysis (see Supplementary Table S7 for details).

*C9orf72* ALS patients (M = 6) showed a median age at disease onset of 58 years (IQR 40.5–67.5). The most frequent ALS phenotypes were bulbar and classic (both 44.4%); only one patient presented with the flail arm variant. All ten cases with expanded C*9orf72* were identified confirming diagnosis made by RP-PCR. Samples als_1, als_2, and als_4 showed very large expansions (als_1 has a mutated reads with an insertion of 3084 GGGGCC). However, several ALS samples showed truncated reads, making impossible to correctly estimate the real expansion size, allowing for only a lower bound. One example of *C9orf72* expansion is shown in Supplementary Fig. S5. ALS cases cannot be confirmed using nanopore coupled with long PCR due to the presence of 100% CGs in the expanded motif. An ALS sample without expansion (als_10) has been confirmed by ONT. Only 1/10 (10%) of C*9orf72* ALS cases showed interruption patterns (als_1 with AGGG). No repeated expansions were found in the gene *LRP12*, which has been reported only in Japanese patients.

A total of 12 out of 46 cases suspected for SCA were detected with at least one STR out of the normal range by native ONT. They showed a median age at disease onset of 64 years (IQR 41–64.9). Cases sc_2 and sc_12 showed unexpectedly an insertion of 120 and 363 ATTCC repeats respectively in *DAB1* (SCA37), since the previously described pathogenic motif was ATTTC. This repeat motif was confirmed by both algorithms (HAC and SUP), and also from long PCR sequencing data. Sample sc_4 had 69 CAG repeats in *ATXN3* (SCA3). A very rare expansion in Caucasian population for *BEAN1* (SCA31) was found in case sc_3 with 431 TGGAA repeats; this case was tested three times both using native and long PCR due to the rarity of this disease in the European population [43] to check possible artefactual results. Case sc_9 showed an expansion in *BEAN1* with 143–161 repeats with mixed TAAAA/TGGAA motifs as well.

Interestingly, case sc_15 was detected to have two loci with pathogenic expansion: *BEAN1*(SCA31) and *FGF14* (SCA27B). Cases sc_5, sc_6, sc_8, sc_13, sc_14, sc_16, and sc_7_cnv_1, showed a repeat expansion in *FGF14* (SCA27B) ranged between 220 and 375 for GAA repeats; the latter (case sc_7_cnv_1) was also found to have both alleles of *RFC1* out of benign range (CANVAS). Among all these cases, no interruption patterns were observed. Five cases showed a single point mutation in *SCN4A* or *CLCN1*. Considering the CANVAS suspected patients, they had a median age at onset of 60.5 years (IQR 57.2–61.2). Nine cases were characterized by homozygosity (cnv_1, cnv_2, cnv_3, cnv_4, cnv_5, cnv_6, cnv_7, cnv_8, cnv_9), while 4 cases (sc_14, sc_1, sc_10, sc_11) showed only one expanded allele. Some cases affected by CANVAS had truncated expanded reads, providing only a lower bound for the estimated size of expansion. Case fxn_1 showed an expansion of 844GAA in one allele, while the other carry the p.(G130V) mutation in *FXN* gene; case fxn_2 revealed both alleles out of benign range.

Sample hd_1 was found to have HD with 47 CAG repeats in *HTT*, while kn_1 was found to have a Kennedy disease with 57 CAG repeats in *AR*. The remaining 29 cases did not show any expansion in the list of investigated loci using AS, as well as for all of the healthy controls.

Expanded samples with SCA, CANVAS, FRDA, HD, and Kennedy, were also tested with nanopore long PCR, Fig. 3A. The Pearson correlation coefficient is 0.557, while the CCC is 0.304. By separating CANVAS from other diseases, however, the agreement between long PCR and native ONT significantly increased, reaching a ρ of 0.986 and a CCC of 0.985, approaching the identity line, Fig. 3B. Instead, although long PCR was successful in detecting *RFC1* expansions in patients with CANVAS, it was unable to accurately determine the size of the expansion (ρ = 0.445 and CCC = 0.143), Fig. 3C.

**Figure 3.**
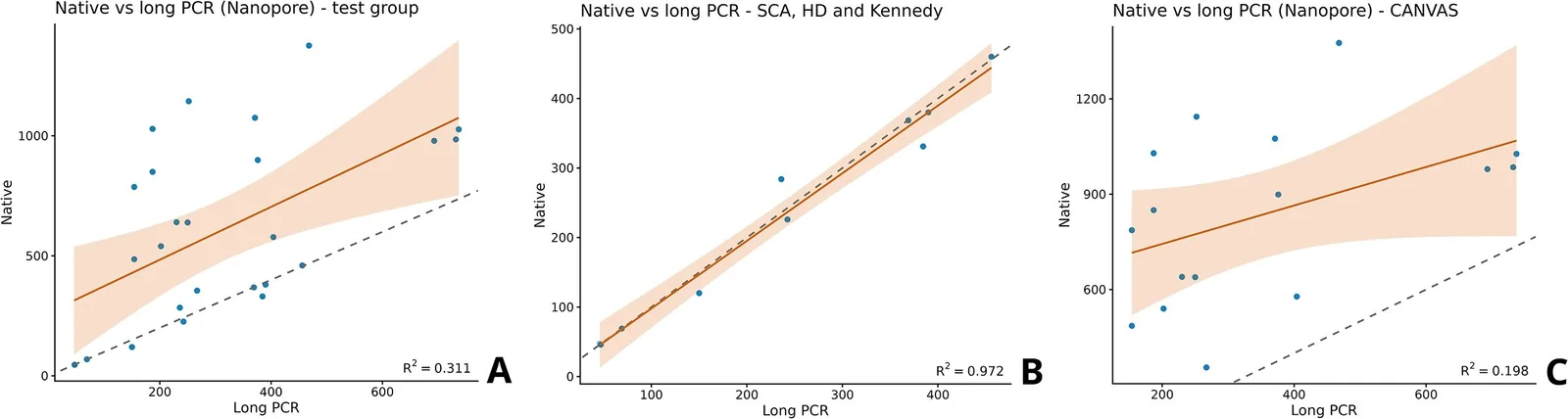
Comparison between native and long PCR nanopore sequencing for test group. (**A**) Scatter plot and linear model for native versus long PCR nanopore estimates of expansions for samples with test group. The 45° line is reported (dashed). The Pearson correlation coefficient is 0.649, while CCC is 0.407. (**B**) Considering only samples with SCA, HD, and Kennedy disease, the agreement significantly improves, as ρ and CCC increases up to 0.976 and 0.975, respectively. (**C**) Samples with CANVAS show a very poor agreement between native and long PCR estimates (ρ = 0.575, CCC = 0.272). Therefore, long PCR could be used as an orthogonal method for ataxias, except for CANVAS.

The complete prospect of diagnoses for the dataset of REDs is reported in Supplementary Tables S7 and S8, and Tables 1 and 2 for DM1, DM2 ALS and test group (SCA, CANVAS, HD, and Kennedy), respectively. Supplementary Table S7 shows the gene of interest, the expansion pattern obtained through NanoExpansion, insertion size estimated from IGV, the reference genome used for the alignment, and the genomic class assigned using SB. Table 1 lists patients tested for ALS, while Table 2 comprises the remaining diseases. For each sample, the target gene (and corresponding RED) is shown, together with IGV expansion size, and reference genome.

**Table 1.** Prospect of the cohort with ALS

ID	Disease (gene)	Expansion pattern	IGV	RP-PCR	Reference
als_1	ALS (*C9orf72*)	GGGGCC (with AGGG)	3; 3084; >1226	expanded	hg38
als_2	ALS (*C9orf72*)	GGGGCC	5; 1421; >470; >445; >312	>163	hg38
als_3	ALS (*C9orf72*)	GGGGCC	8; >62	>145	hg38
als_4	ALS (*C9orf72*)	GGGGCC	8; 1331	>166	hg38
als_5	ALS (*C9orf72*)	GGGGCC	2; >587	>154	T2T
als_6	ALS (*C9orf72*)	GGGGCC	2; >589; >497; >340	expanded	T2T
als_7	ALS (*C9orf72*)	GGGGCC	2;>33	expanded	T2T
als_8	ALS (*C9orf72*)	GGGGCC	5; >32	expanded	hg38
als_9	ALS (*C9orf72*)	GGGGCC	25; 26	25 (PRE*)	T2T
als_10	ALS (*C9orf72*)	GGGGCC	5; 8	5 (NE**)	hg38
als_11	ALS (*C9orf72*)	GGGGCC	52; 77	59	T2T

Repeat expansion patterns characterization for ALS patients in gene *C9orf72*. IGV visual inspection (native ONT), RP-PCR estimate, and reference genome are reported. Usually, native ONT cannot sequence the entire expanded reads, resulting in a lower limit for the expansion size. Agreement between ONT and RP-PCR is good for pre-expanded and nonexpanded samples (als_9, als_10, and als_11).

*pre-expanded. **notexpanded.

**Table 2.** Prospect of patients of the test group

ID	Disease (gene)	Expansion pattern	Repeats with IGV (native)	Repeats with IGV (long PCR)	Reference
sc_2	SCA37 (*DAB1*)	ATTCC	18; 120	18; 150	hg38
sc_3	SCA31 (*BEAN1*)	TGGAA	34; 434–460	34; 448–464	hg38
sc_4	SCA3 (*ATXN3*)	CAG	7; 67–69	14; 69	hg38
sc_5	SCA27B (*FGF14*)	GAA	7; 340–375	7; 363–375	hg38
sc_6	SCA27B (*FGF14*)	GAA	11; 220–230	11; 229–256	hg38
sc_7, cnv_1	SCA27B (*FGF14*) CANVAS (*RFC1*)	GAA AAGGG	77; 284 967; 1144	77; 236 250–252	hg38
sc_8	SCA27B (*FGF14*)	GAA	10; 302–331	10; 371–398	hg38
sc_13	SCA27B (*FGF14*)	GAA	42; 382	42; 304–382	hg38
sc_14	SCA27B (*FGF14*)	GAA	25; 254	25; 220–260	T2T
sc_15	SCA27B (*FGF14*) SCA31 (*BEAN1*)	GAA TAAAA/AATGG	*FGF14:* 16; 479, 476, 488, 482, 477; *BEAN1*: 11; 353, 293, 352, 331, 356, 350, 416, 349, 340	*FGF14:* 16; 476–488 *BEAN1*: 11; 293–416	T2T
sc_16	SCA27B (*FGF14*)	GAA	5; 343	5; 343	T2T
sc_9	SCA31 (*BEAN1*)	TAAAA/AATGG	11; 143–161(TAAAA/AATGG)	11; 163	hg38
sc_12	SCA37 (*DAB1*)	AAAAT/AATGG	79; 125AAAAT163AAT GG92AAAAT	79; 119AAAAT;128 AATGG	hg38
cnv_2	CANVAS (*RFC1*)	AAGGG	434–486; 985	154; 732	hg38
cnv_3	CANVAS (*RFC1*)	AAGGG	640; 873–979	230; 693	T2T
cnv_4	CANVAS (*RFC1*)	AAGGG	540; 1027	202; 737	T2T
cnv_5	CANVAS (*RFC1*)	AAGGG	1029; 1075	187; 371	T2T
cnv_6	CANVAS (*RFC1*)	AAGGG	850; 908	187; 376	T2T
cnv_7	CANVAS (*RFC1*)	AAGGG	355, 578	267; 404	T2T
cnv_8	CANVAS (*RFC1*)	AAGGG	1376; 1284; 1119; 928; 820; 814; 810; 808; 803; 787	154; 468	T2T
sc_14	CANVAS (*RFC1*)	AAAAG/AAGGG	10; 582	10; 564	hg38
sc_1	CANVAS (*RFC1*)	AAAAG/AAGGG	126; 855	126; na	hg38
sc_10	CANVAS (*RFC1*)	AAAAG/AAGGG	111; 940	111; na	hg38
sc_11	CANVAS (*RFC1*)	AAAAG/AAGGG	113; 992	113; na	hg38
cnv_9	CANVAS (*RFC1*)	AAAAG/AAGGG	1151; 1515	NA	T2T
hd_1	HD (*HTT*)	CAG	47; 45	46	hg38
kn_1	Kennedy (*AR*)	CAG	55–57	57	T2T
fxn_1	Friedreich (*FXN)*	GAA	844/p.G130V	844	T2T
fxn_2	Friedreich (*FXN)*	GAA	693; 743	693; 743	T2T

Repeat expansion patterns characterization for SCA, CANVAS, HD, Kennedy disease, and FRDA patients. Disease (with corresponding gene), expansion motif, IGV visual inspection (both native and long PCR ONT), and genome reference are reported. For some CANVAS patients, only a lower limit is given. In general, the expansion size estimates for patients with SCA, HD, and Kennedy was detected to be stable.

For some patients, the mutated reads significantly differed in terms of length and composition, showing the presence of the mosaicism phenomenon [44]. For the cohorts of patients listed in Supplementary Table S7, 1 and 2, several structural variant caller tools (Straglr, Sniffles2, CuteSV, and NanoRepeat) were compared, together with visual inspections from IGV, to establish which was the most suitable for being incorporated in the LRS based diagnostic pipeline. Supplementary Tables S9–S12 report all the estimated size obtained through the different variant callers, for DM1 (hg38 and T2T), ALS, and test group, respectively. A graphical comparison between results from different tools and IGV (taken as ground truth) are shown in Fig. 4 and Supplementary Fig. S6. From both Bland–Altman plots (Supplementary Fig. S6A–H) and violin plots (Fig. 4), Straglr and NanoExpansion (where available) consistently outperformed the other software tools. All the computed metrics shown in Supplementary Table S13 confirmed this result. Benchmarking of NanoExpansion is detailed in Supplementary material.

**Figure 4.**
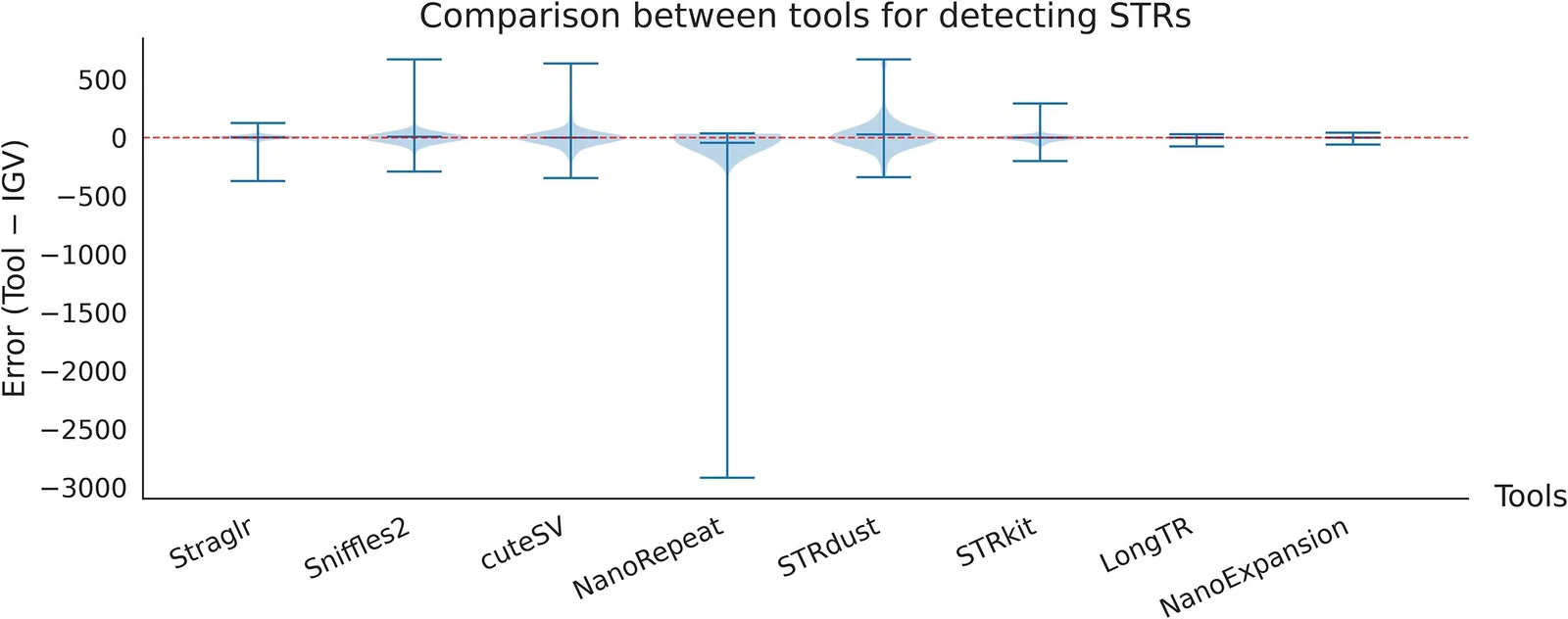
Comparison between NanoExpansion and the other variant caller software tools. Violin plots of the distribution of errors (tool estimate – IGV estimate) for all the variant callers (Straglr, Sniffles2, CuteSV, NanoRepeat, STRdust, STRkit, LongTR, and NanoExpansion). LongTR, and NanoExpansion show smaller errors with respect to the remaining softwares. NanoRepeat tends to underestimate the expansion size.

Supplementary Table S14 summarize the comparison between the number of repeats recognized by short read sequencing (exome sequencing with NovaSEQ6000) and ONT. A perfect agreement between the two approaches was achieved for wild type alleles, when short read sequencing was able to characterize expansion numbers covering the whole locus with repeat motifs. However, when the number of repeats exceeded 150 bp, NovaSEQ6000 gave no informative data due to short sequencing reads. Expansion size estimates were done through visual inspection using IGV, consistently with the approach used for long reads. Underestimated expansion size or inferior limits were obtained.

## Discussion

The use of ONT sequencing coupled with AS is crucial in designing an advanced diagnostic tool for REDs. New genomic regions associated with these diseases can be easily included in the workflow, by simply adding the new coordinates to the BED file used for the enrichment. It is essential to include the Dorado sup model in the bioinformatic workflow to achieve more robust, high-quality results (Fig. 1A), even though basecalling takes longer than with the hac model. In addition, the use of AS is another crucial aspect of pipeline design, since it enables consistent enrichment of the region of interest, as confirmed by ER and FE. An FE >4× (and ER above 5×) demonstrated the efficacy of AS. The relatively high internal variability could be explained by the fact that some samples underwent AS with a minor number of target regions, resulting in underestimated enrichment metrics. Moreover, the different amounts of starting DNA could have also affected the AS enrichment efficacy. Exploiting T2T reference improved the mapping quality of samples (see Fig. 1B). Moreover, since it has been constructed using long read techniques, it would allow to better estimate the expansion detection and sizes.

Currently, with our High-Performance Computing (HPC) infrastructure, each sample required ∼10 h of computational time to perform super accurate basecalling with base modifications (5mCG–5hmCG). However, it is important to notice that the computational time is affected by the amount of starting DNA. A run with a mean coverage of 10–15× requires about 10 h. Our HPC setup enables the concurrent processing of two samples in parallel. The GridION platform, which is GPU enabled, can run an additional super accurate basecalling (in ∼20 h). The downstream integrative analysis of each sample is then completed in ∼2 h. The possibility to use hac or fast basecalling and selecting only part of downstream analysis (according to the need of the user) can significantly drop the run time and computational overhead (especially selecting fast basecalling, which however does not allow for base modification detection). Also running sup basecalling without base modification would halve the total computational time.

Focusing on the DM1 cohort, all individuals were correctly found to be expanded (detecting >50 CTG expansion), confirming the SB diagnosis and clinical data. Considering the assigned genomic class, ONT correctly classified 28 subjects, while six were misclassified. The agreement between SB and ONT increases when considering the statistical metrics. In particular, the median of coverage ratio and distance to confidence interval, which estimates the overlapping of ONT and SB, were optimal, proving the reliability of ONT in quantifying the repeat expansion size. The relatively high widths of uncertainties estimates are due to the high skewness of the distribution (majority of cases in agreement, a few that are discordant).

The benefits of exploiting ONT sequencing for the characterization of expanded ALS samples for *C9orf72* are shown in Supplementary Fig. S5. The large GGGGCC hexanucleotide expansion (up to 3084 and 1421 repeats, for case als_1 and als_2, respectively) requires ultra long reads to be fully captured, since RP-PCR cannot completely characterize it. The use of sup basecalling allowed to correctly characterize repeat expansions in all investigated ALS cases, minimizing false repeat interruption calls. In this small cohort, no repeated expansions were found in the gene *LRP12*, which has been reported only in Japanese patients [45, 46]. We detected >30 expansion repeats in all cases except one revealing 26 repeats, confirming data from RP-PCR. Since RP-PCR provided only a lower limit for the expansion size, we did not compute any agreement metrics between ONT and RP-PCR estimates.

The implementation of ONT LRS in the diagnostic process for detecting STRs in neurological disorders represents a valuable tool for achieving accurate diagnosis more quickly and efficiently [3]. For example, >40 known dominant SCA syndromes present with cerebellar ataxia with overlapping features, makes clinical differentiation almost impossible without genetic testing. Traditional approaches require sequential testing of individual genes, which can take months and may still miss rare variants. The ability to make an accurate differential diagnosis through a single laboratory test can facilitate the early provision of genetic counselling with an accurate description of disease severity and prognosis. This could avoid the slow, labor-intensive, and resource-intensive methods traditionally required for STR detection across multiple genes, such as SB or RP-PCR. Early recognition of similar genetic diseases will become particularly valuable for patient enrolment in clinical trials and with the emergence of new disease-modifying treatments, where early administration is typically associated with better outcomes.

The entire cohort of suspected SCA patients was composed of 46 individuals, of which only 12 showed repeat expansions in the known regions of interest (two in two loci). All these cases were also evaluated for exome sequencing, finding mutations in 5 non expanded cases in *SCN4A* or *CLCN1* genes. The remaining 29 cases did not show any expansion in the list of investigated loci using AS. This may depend on the fact that not all the forms of SCA are characterized by an STRs, even though symptoms are similar among different forms.

Long PCR validated results obtained with native ONT, however, while the estimates for shorter expansions are very accurate, it underestimated expansions larger than 800 repeats. This is expected, since PCR failed to amplify very large reads especially with GC-rich regions. Moreover, long PCR provides reads with expansions varying in size (see Supplementary Fig. S3), since it generated a high number of reads derived from many cells which may be affected by mosaicisms. For this reason, it is important to note that long PCR can be adopted as a valid alternative to native ONT, providing an orthogonal method of assessing native ONT results at limited additional cost. One limitation of this approach is the generation of incomplete reads due to partial polymerization of the region of interest by polymerase enzyme during the PCR process. However, these artefacts can be easily detected and excluded from the analysis. We considered only reads that cover the repeats and the flanking regions including primers. Correlation plots confirmed the agreement between native and long PCR ONT estimates, highlighting the problem of large expansions. Long PCR resulted to be very accurate in detecting expansions for SCAs, reaching a CCC of 0.975 (see Fig. 3B). However, considering samples with CANVAS, the agreement resulted to be very poor, probably due to the length of expansions in *RFC1* in GC-rich region.

In terms of identifying repeat expansions, IGV was used to inspect the repeat patterns and validate results from other tools. In our hands, LongTR resulted to be the most performant software with respect to Straglr, Sniffles, CuteSV, NanoRepeat, STRdust, and STRkit. Nevertheless, none of these tools provided a comprehensive description of the composition of the repeat patterns. Although IGV allowed direct visualization of repeat composition in both native and long-PCR datasets, this process required manual inspection and interpretation, making it impractical for routine analyses or large-scale studies. NanoExpansion was developed to specifically address this limitation by generating a compact representation of repeat patterns, while simultaneously extracting methylation information. Although LongTR had a lower MAE in repeat size estimation, NanoExpansion demonstrated superior overall performance when the HR was considered, demonstrating greater robustness in scenarios resembling routine diagnostic sequencing (reduced coverage).

Across all evaluated software, some repeat expansions were missed in individual patients. This might be due to technical challenges, including partially aligned reads, incomplete reads terminating within the expanded repeat tract, or sequencing errors that complicate repeat boundary identification, which can alter the performance of the variant detection tools. NanoExpansion consistently reconstruct the repeat patterns in a larger portion of samples, outperforming all the other evaluated tools (Fig. 4 and Supplementary Fig. S6), additionally providing information on interruption motifs and methylation patterns. Moreover, the algorithm is relatively simple and operates on a restricted subset of reads spanning the locus of interest, avoiding computationally expensive analyses. The analysis of a single sample can be completed in <1 min on a standard desktop computer, making NanoExpansion suitable for both research applications and routine laboratory workflows.

As shown in Fig. 2, the CTG repeat size is variable in cells belonging to the same specimen. This phenomenon is called mosaicism and is known to characterize several REDs [44]. CTG instability in DM1 patients can be present in both somatic and germline cells and can change over time [44]. The mosaicism condition is known to be associated with patient prognosis. A decrease in the somatic mosaicism is often associated with a less severe clinical phenotype [12], confirming the importance of characterizing repeat expansion patterns. Mosaicism forms, when found in native DNA, were also confirmed in long-PCR data analysis. In addition, given the high stability of expansion estimates for patients with SCA, HD, and Kennedy disease (see Table 2), it is highly likely that the variability observed in some patients of the DM1 cohort (Supplementary Table S7) raised from mosaicism, rather than artefactual errors.

The inspection of interruption insertions is another crucial aspect of this diagnostic pipeline. It is known that CTG expansions in *DMPK* gene can be characterized by the presence of alternate motifs. The effects of these interruption patterns are not yet fully understood. For instance, Swinkels H *et al*. [47] reported that intermediate repeats of size 37–43 with CCGCTG interruptions are not prone to further expansion, and therefore they do not act as pre-mutations. An accurate characterization of them is crucial for prognostic purposes. Since ONT is prone to sequencing errors, we observed how an interruption motif changed according to the basecalling model. In particular, the TTG motif, the most frequently inserted interruption, always disappeared after re-basecalling with the sup model. CCG appeared to be more accurate as it was consistently identified by both basecalling models. Moreover, the presence of CCG motif in two individuals (dm_6 and dm_10) is observed also using long PCR, confirming what was found with native nanopore sequencing. When a CTC interruption is detected, other expanded reads from the same samples showed only noninterrupted CTG motifs (Supplementary Table S7). This fact could indicate that these CTC interruptions could be mosaicisms due to the high coverage depth potential to detect variants, or alternatively possible artefacts, due to the inherent inaccuracies of the sequencing techniques. The fact that the interruption pattern was only detected in DM1-expanded cases and not in the other loci investigated (with one exception for one *C9orf72* expanded case), suggests the high accuracy of nanopore sequencing in limiting the generation of artefactual results.

Some limitations need consideration for the present study. First, the patient cohort is relatively small, since REDs are rare diseases. However, due to the continuous patient enrolling at our center, the size of the cohort is expected to increase. Indeed, this diagnostic workflow should be further validated on a larger dataset, and we are currently planning a multicentre study to test its robustness and scalability. Furthermore, the use of high throughput PromethION platform (ONT) will increase the coverage depth, related accuracy and precision.

In summary, the diagnostic pipeline based on both native DNA and long PCR sequencing allows to correctly detect and characterize STRs. Although NanoExpansion proved to be a valuable component of the analysis pipeline, the current implementation still has some limitations, which will be addressed in further development. First, its performance should be validated on additional datasets, possibly generated in different experimental conditions, to assess the robustness and generalizability of the algorithm. This step is crucial before integrating NanoExpansion into routine clinical bioinformatic workflows. Additional developments also include improved handling of highly fragmented reads, and a more efficient automatic motif detection module, to enable the reveal of previously unreported interruption patterns. Despite these limitations, NanoExpansion provides a comprehensive framework for the analysis and characterization of pathogenic repeat expansions and represents a promising foundation for future methodological developments.

Finally, the patients belonging to this research cohort were undergoing clinical examinations, magnetic resonance imaging, and neuropsychological evaluation. This is a crucial aspect, since collecting these different sources of information would allow to correlate them with the genomic data. Integrating multi-modal data and exploiting statistical methods and machine learning algorithms would help to shed light on the genotype–phenotype correlation, which remains an open question with regard to these REDs.

This study considered a relatively large cohort of repeat expansion patients, which proved the robustness of the proposed diagnostic pipeline. This could be further strengthened by applying the pipeline to other STR datasets. The pipeline could potentially become standard practice for diagnosing patients at the IRCCS Istituto delle Scienze Neurologiche di Bologna and other healthcare institutions.

## Supplementary Material

lqag117_Supplemental_File

## Data Availability

All sequenced samples are deposited in NCBI Sequence Read Archive (https://submit.ncbi.nlm.nih.gov/subs/sra/) under BioProject ID: PRJNA1526302. The Sample ID accession numbers correspond to those listed in Tables 1 and 2 and Supplementary Table 7. The Snakemake pipeline used to run the bioinformatic workflow presented in this article can be found at https://zenodo.org/records/18619310, except for the NanoRepeat tool, which can be directly downloaded from its Zenodo repository. The implementation of NanoExpansion can be found at https://doi.org/10.5281/zenodo.21675564. All the figures were generated using popular Python (Matplotlib and Seaborn) and R (ggplot2) plot libraries, except for IGV screenshots.
