# Supplementary material for: Nanopore sequencing combined with adaptive sampling and NanoExpansion enables accurate characterization of repeat expansion disorders
Source: NAR Genom Bioinform. 2026 Oct 6;8(4):lqag117. doi: 10.1093/nargab/lqag117 (PMC13639371; doi:10.1093/nargab/lqag117)
Supplement: Supplementary file 1 [file lqag117_supplemental_file.pdf]

Supplementary File 1

Primers details used for long-PCR sequencing

Primers for PCR were designed using *Primer3*program (https://primer3.ut.ee). We obtained forward and reverse primers for 19 genes, causative for 19 different repeat expansion disorders. The region of interest of each gene comprises the repeated motif and approximately 20-30 surrounding bases. These regions have been identified on *Ensembl* genome browser and, subsequently, it has been inserted into *Primer3* as template sequence where the left and the right primers are picked. We inserted the following parameters:

	Min.	Opt.	Max.
**Primer size:**	18 bp	20 bp	30 bp
**Primer melting temperature (Tm):**	67°C	72°C	72°C
**Product size range:**		200-8000 bp	

Amplicons were checked on *UNAfold* web server (https://www.unafold.org/mfold/applications/dna-folding-form.php), to ensure that they do not form hairpins, which would reduce their annealing ability. Additionally, the whole amplicon sequences were checked on *RepeatMasker* (https://repeatmasker.org), to screen DNA sequences for interspersed repeats. Finally, in-silico PCR on UCSC genome browserhas been performed to check the quality of the primers and whether the STR region is comprised or not. The genes amplified with long PCR and their respective primers are reported in the following Tables:

DMPK (DM1)

	Sequence	Tm (°C)	Amplicon size
Left primer	CCTGCTCCTGTTCGCCGTTGTTCTGTCTCG	72.01	924 bp
Right primer	ATTTCCCGAGTAAGCAGGCAGAGATCGCG C	71.68	

CNBP (DM2)

	Sequence	Tm (°C)	Amplicon size
Left primer	TCCACAAACTCCCTCAAATACCAAAATCCTCC T	67.37	1942 bp
Right primer	CCACGGCTTCTCATTCCACGACCAC	67.65	

AR (Kennedy disease)

	Sequence	Tm (°C)	Amplicon size
Left primer	ATCTTGTCCACCGTGTGTCTTCTTCTGCAC	68.46	942 bp
Right primer	GGCGTTGTCAGAAATGGTCGAAGTGCCCC	71.26	

C9ORF72 (ALS)

	Sequence	Tm (°C)	Amplicon size
Left primer	CCCACCCTCTCTCCCCACTACTTGCTCTCA	71.27	854 bp
Right primer	ACCCAGCTTCGGTCAGAGAAATGAGAGGGA	69.96	

ATXN1 (SCA1)

	Sequence	Tm (°C)	Amplicon size
Left primer	GCTCACCTGCCGCACACCTTCCAGTTCATT	72.20	1280 bp
Right primer	GTGAGCGTGTGTGGGATCATCGTCTGGTGG	71.76	

ATXN2 (SCA2)

	Sequence	Tm (°C)	Amplicon size
Left primer	CGCCTCCCTCCGCCTCAGACTGTTTTGGTA	72.50	1029 bp
Right primer	ACCCCTCCAACGTTCACACCTAAACCGGGA	72.13	

ATXN3 (SCA3)

	Sequence	Tm (°C)	Amplicon size
Left primer	TGGGATGGTCAGGAAAGGCATCTCTGGGGA	71.61	3979 bp
Right primer	ACTGTGGTTTTATCGTGCATTTCCCTGTGACT	68.23	

CACNA1A (SCA6)

	Sequence	Tm (°C)	Amplicon size
Left primer	CCTCCCAATCCCGTGTCTCCTTTGATTTTGT	68.37	2314 bp
Right primer	GTCACTCTCGCTGTAGGGTTCGTGCA	68.28	

ATXN7 (SCA7)

	Sequence	Tm (°C)	Amplicon size
Left primer	GGACGAATTCAAGTTCCCCAGGCGCACAGT	71.99	6639 bp
Right primer	TCGGGGAGTCGGTATGGTGGGCTGATGTTC	72.23	

TBP (SCA17)

	Sequence	Tm (°C)	Amplicon size
Left primer	TGCACACCTGACCTGCTGTTCCACCAAGAA	71.37	3606 bp
Right primer	CACAATTACCGTTAGTGCCACTCCCTCCCT	69.29	

NOP56 (SCA36)

	Sequence	Tm (°C)	Amplicon size
Left primer	GCTCTGCTGCCCGTAACTCATATGACAAGA	68.05	1247 bp
Right primer	CCCGGATCTAGAGCTTTCCAGGCCCGTCC	72.67	

LRP12 (ALS2)

	Sequence	Tm (°C)	Amplicon size
Left primer	AGTCCACACTGCCCCTTGACCGACCAG	71.52	1281 bp
Right primer	CCTCAGCTTTGTTCGGTCAGCGGGGCG	73.10	

FMR1 (FRAXA)

	Sequence	Tm (°C)	Amplicon size
Left primer	CGTGGGTCAAAGCTGGGTCTGAGGAAAGGC	72.01	4167 bp
Right primer	ATGCACTGCTGGGCCATGTTAGGGTCTTCC	71.64	

RFC1 (CANVAS)

	Sequence	Tm (°C)	Amplicon size
Left primer	TCAAGTGATACTCCAGCTACACCGTTGC	69.10	354 bp
Right primer	AGCATTGTGGGAGACAGGCCAATCA	71.70	

FXN (Friedrich's ataxia)

	Sequence	Tm (°C)	Amplicon size
Left primer	CGTCCAAGCCATATTTGTGTTGCTCTCCGG	69.37	900 bp
Right primer	CGACACCACGCCCGGCTAACTTTTCTTTAT	68.93	

FGF14 (SCA27)

	Sequence	Tm (°C)	Amplicon size
Left primer	TGATGAATTAAGCAGTTCCTGCCCACA	70.20	537 bp
Right primer	GAGCGGTTGTCAGAATTTCAGGTCCA	70.20	

DAB1 (SCA37)

	Sequence	Tm (°C)	Amplicon size
Left primer	TGGCTGAAGTGGTCCTCCCAAGTCA	72.00	418 bp
Right primer	ACACAGCCAGCAAAGGACAGCTGGA	72.10	

BEAN1 (SCA31)

	Sequence	Tm (°C)	Amplicon size
Left primer	TGGGCCAAGATCACGTCACTGCAC	67.43	1440 bp
Right primer	TGGGCCTGGGGATTGGGACCTGCT	71.74	

HTT (Huntington's disease)

	Sequence	Tm (°C)	Amplicon size
Left primer	ATGGCGACCCTGGAAAAGCTGATGAAGGCC	72.00	650 bp
Right primer	GGCTCTGGGTTGCTGGGTCACTCTGTCTCT	72.16	

PCR conditions:

Long PCR was performed following the user guide for Phusion U Hot Start DNA Polymerase. The reaction mix was prepared by adding H2O (8 µl), 5X Phusion GC Buffer (4 µl), Betaine 5M (4 µl), dNTPs 10 mM (0,4 µl), Phusion U polymerase (0,2 µl), Primer mix 5 pmoles (1,6 µl), DNA sample (2 µl). The use of GC buffer improves the performance of the polymerase in GC-rich regions and in long templates with complex secondary structures.

The following long PCR amplification program was used:

1) Initial activation step: 95°C for 1 minute 45 seconds.
2) 30 cycles:
- Denaturation: 98°C for 20 seconds,
- Annealing: 68°C for 30 seconds,
- Extension: 72°C for 8 minutes.
3) Final step: 72°C for 5 minutes

Benchmarking of NanoExpansion

The benchmarking of NanoExpansion has been conducted in the following way: 11 reads have been synthetically manually generated using Seq package (Python), inserting different expansion patterns (known by the user). The selected use-case was a DM1 sample. The choice was because we detected interruptions only in DM1 individuals.

In the following table, the original patterns (ground truth) and the ones detected and outputted by NanoExpansion are reported. As can be clearly seen, NanoExpansion succeeded in characterizing each pattern. The only difference is that NanoExpansion only reported patterns in the form (motif)N for main motif and main interruptions. We chose to test only files with a single synthetically generated read because NanoExpansion analyzes each read separately (there is no difference in using a BAM with multiple expanded reads).

However, two main limitations affect NanoExpansion: the first is that it is based on Straglr. Therefore, if an expansion is not detected by Straglr, the same happens for NanoExpansion. Moreover, the software can correctly characterize only repeat patterns starting and ending with the same motif (e.g., (CTG)N...(CTG)M), while it fails if this condition is not met (see sample c in the following table); however, a different condition cannot be found (also Straglr cannot detect similar cases).

The NanoExpansion benchmarking can be executed from the software repository, where the synthetic files are present.

**Sample**	**Ground truth**	**NanoExpansion output**
a	(CTG)295(CA)2(CTG)132	(CTG)295(ACA)(CTG)132
b	(CTG)265(CGG)193(CTG)1112	(CTG)265(CGG)193(CTG)1112
c	(CTG)25(CCG)21(CTG)58(CCG)14	(CTG)25(CCG)21(CTG)58(CCG)14
d	(CTG)32(CTC)410(CTG)34	(CTG)32(CTC)410(CTG)34
e	(CTG)31(CCG)57(CTG)33	(CTG)31(CCG)57(CTG)33
f	(CTG)159(CCG)5(CTG)1(CCG)1(CAG)1(CCG)3(CTG)23	(CTG)159(CCG)5(CTGCCGCAG)(CCG)3(CTG)23
g	(CTG)30(CG)1(CA)1(CTG)20(CCG)1(CT)1(CTC)10(CTG)20	(CTG)30(CGCA)(CTG)20(CCG)(CTC)10(CT)(CTG)20
h	(CTG)37(CAC)1(CCG)2(CTC)12(CTG)48	(CTG)37(CAC)(CCG)2(CTC)12(CTG)48
i	(CTG)1249	(CTG)1249
j	(CTG)29(CCG)3(CTG)15(CCG)7(CTG)56	(CTG)29(CCG)3(CTG)15(CCG)7(CTG)56
k	(CTG)341(CCG)3(CA)1(CCG)9(CTG)1317	(CTG)341(CCG)3(CA)(CCG)9(CTG)1317

Definition of metrics used in statistical analysis

The comparisonbetweenhac and supbasecalling models (and alignment to hg38 and T2T referencegenome) hasbeendone by computing the following error rates:

$$mismatchrate=\frac{\#mismatchbases}{\#alignedbases}$$

$$insertionrate=\frac{\#insertedbases}{\#alignedbases}$$

$$deletionrate=\frac{\#deletedbases}{\#alignedbases}$$

$$Homopolymererrorrate=\frac{\#errors \in homopolymers}{\#bases \in homopolymers}$$

$$Editdistance=N_{mismatch}+N_{insertion}+N_{deletion}$$

$$MAPQ=-10log_{10}(P_{misalignment})$$

$$Clippedbases=N_{soft}+N_{hard}$$

In order to compare the effectiveness of AS, the foldisdefinedas:

$$f=\frac{ON\text{-}TARGETbases}{TOTALbases}$$

Therefore, the ER and FE are definedas:

$$ER=\frac{f_{AS}}{\frac{targetdimension}{referencegenomedimension}}$$

$$FE=\frac{f_{AS}}{f_{control}}$$

An FE greater than 4x and an ER above 5x can demonstrate the efficacy of AS.

To compare SB and native ONT results, weusedCohen's Kappa, ordinalaccuracy, coverage rate, distance of the median from interval, and probabilisticoverlap:

$$\kappa_w=\frac{1-\sum_{ij} w_{ij}O_{ij}}{1-\sum_{ij} w_{ij}E_{ij}}, \text{ with } w_{ij}=\frac{(i-j)^2}{(k-1)^2}$$

$$Ordinal accuracy=\frac{1}{N}\sum_{i=1}^{N} 1\left(\left|\overline{y}_i\text{-}y_i^{\mathrm{GT}}\right| \leq 1\right)$$

$$coverage\text{-}rate_i=\frac{1}{N}\sum_{i=1}^{N} 1\left(y_i^{\mathrm{GT}} \in [L_i, U_i]\right)$$

$$d_i=0 if m_i \in [L_i, U_i], else\{min(|m_i\text{-}L_i|, |m_i\text{-}U_i|)\}, \text{ where } m_i=median(y_i)$$

$$overlap_i=\int min\left(p_A(x), p_B(x)\right)dx$$

where O_{ij} and E_{ij} are observed and expected frequencies for cell ij (confusion matrix), y_i^{GT} is the ground truth, $\overline{y}_i$ the predicted value, L_i and U_i lower and upper bound respectively, and $p_A(x), p_B(x)$are the kernel density estimates.
To compare results obtained with the different variant caller software tools, the following metrics have been used:

$$MAE=\frac{1}{N}\sum_{\mathrm{i=1}}^{\mathrm{N}}|e_i|$$

$$RMSE=\sqrt{\frac{1}{N}\sum_{\mathrm{i=1}}^{\mathrm{N}} e_i^2}$$

$$Bias=\frac{1}{N}\sum_{\mathrm{i=1}}^{\mathrm{N}} e_i$$

$$CV=\frac{\sigma}{\mu}$$

Where$e_i=\overline{x}_i\text{-}x_i$ is the absolute error.

The hit rate (HR) is defined as the ratio between the number of calls made by the tool and the number of calls observed using IGV (ground truth).

$$HR = \frac{\#ofcallsmadebythetool}{\#callsobserved(groundtruth)}$$

The combined score (CS) that considers both accuracy and HR is defined as:

$$CS = \frac{HR}{1 + MAE}$$

The Intraclass Correlation Coefficient (ICC) and the Lin's Concordance Correlation Coefficient (CCC) are defined as:

$$ICC = \frac{\sigma^2_{between}}{\sigma^2_{between} + \sigma^2_{within}}$$

$$CCC = \frac{2\rho\sigma_x\sigma_y}{\sigma^2_x + \sigma^2_y + (\mu_x - \mu_y)^2}$$

where $\sigma^2_{between}$ and σ^2_{within} are variance between groups and within groups, respectively, while $2\rho\sigma_x\sigma_y$ is the covariance of measures x and y (σ^2_x, σ^2_y and μ_x, μ_y are their variance and mean, respectively).

Supplementary Table 1: Target loci (hg38) for adaptive sampling. List of target loci used for the AS, including chromosome, gene of interest, dominance, disease, location within the gene, precise hg38 coordinate, number of repeats (hg38), normal range, and pathological range.

Chromosome	Gene	Dominance	Disease	Location/direction	Precise coordinate	Number of repeats in hg38	Normal range	Pathological range
chr19:45,769,717-45,782,490	*DMPK*	AD	DM1	3'UTR/-	chr19:45770200	20CTG	5-37	>50-10000
chr3:129,167,827-129,183,896	*CNBP*	AD	DM2	intron/-	chr3:129172650	(CAGG)7CAGACAGGCAGACAGGCAGC(CAGG)8(CAGA)10	11-30	>50
chrX:67,544,021-67,730,619	*AR*	XLR	Kennedy disease	coding/+	chrX:67545320	22CAG+CAA(23Q)	9-36	>38-68
chr9:27,546,546-27,573,481	*c9orf72*	AD	ALS	5'UTR intron/-	chr9:27573523	3GGGGCC	3-25	>25
chr6:16,299,112-16,761,460	*ATXN1*	AD	SCA1	coding/-	chr6:16327700	29CAG	6-38	>39-88
chr12:111,452,214-111,599,315	*ATXN2*	AD	SCA2	coding/-	chr12:111599000	23CAG	13-31	>32-500
chr14:92,058,552-92,106,582	*ATXN3*	AD	SCA3	coding/-	chr14:92071010	14CAG	12-44	>55-887
chr19:13,206,442-13,506,479	*CACNA1A*	AD	SCA6	coding/-	chr19:13207883	13CAG	4-18	>20-33
chr3:63,863,867-64,003,462	*ATXN7*	AD	SCA7	coding/+	chr3:63912707	10CAG	4-33	>37-460
chr6:170,554,369-170,572,859	*TBP*	AD	SCA17	coding/+	chr6:170561947	38CAG(or CAG/CAA)	25-40	>43-66
chr20:2,652,632-2,658,393	*NOP56*	AD	SCA36	intron/+	chr20:2652740	4GGCCTG	5-14	>650-2500
chr8:104,489,236-104,589,258	*LRP12*	AD	ALS2	5'UTR/-	chr8:104588983	12CGG	13-45	>90-130
chrX:147,911,919-147,951,125	*FMR1*	XLR	FRAXA	5'UTR/+	chrX:147912082	20CGG	5-50	>200
chr4:39,287,456-39,366,362	*RFC1*	AR	CANVAS	intron/-	chr4:39,348,420	12AAAAG	variable	>400AAGGG
chr9:69,035,752-69,079,076	*FXN*	AR	Friedrich's ataxia	intron/+	chr9:69037285	6GAA	5-34	>66-1300
chr13:101,708,804-102,404,443	*FGF14*	AD	SCA27	intron/-	chr13:102161575	50GAA	5-250	>250
chr1:56,994,778-57,424,060	*DAB1*	AD	SCA37	intron/-	chr1:57367024	17ATTTT	7-400(ATTTT)	ATTTC>31-75
chr16:66,427,295-66,482,833	*BEAN1*	AD	SCA31	intron/+	chr16:66490397	13TAAAA	variable	TGGAA>110(+TAGAA)
chr22:45,671,834-45,845,307	*ATXN10*	AD	SCA10	intron/+	chr22:45819146	14ATTCT/ATTGT	10-32	>280
chr5:146,580,742-146,878,757	*PPP2R2B*	AD	SCA12	5'UTR/-	chr5:146878727	11CAG	4-32	>43
chr13:70,099,727-70,157,682	*ATXN8/ATXN8OS*	AD	SCA8	3'UTR/+	chr13:70139400	CAG/CTG_11ACT_15GCT	15-50	>74-250
chr4:3,074,681-3,243,957	*HTT*	AD	HTT	coding/+	chr4:3074904	21CAG	6-35	>36-250
chr12:6922459-6944321	*ATN1*	AD	DRPLA	coding/+	chr12:6936750	19CAG	3-35	48-93
chr16:72,782,885-73,048,128	*ZFHX3*	AD	SCA4	coding/+	chr16:72787695	21GGC	14-26	>42-74
chr21:43,773,950-43,776,308	*CSTB*	AR	Unverricht-Lundborg (ULD)	5'UTR/-	chr21:43776462	6CCCCGCCCCGCCG	2-3	>30
chr16:67,842,320-67,844,195	*THAP11*	AD	SCA	coding/-	chr16:67842894	29CAG	20-38	>45-100

Supplementary Table 2: Target loci (T2T) for adaptive sampling. List of target loci used for the AS, including chromosome, gene of interest, dominance, disease, location within the gene, precise T2T coordinate, number of repeats (T2T), normal range, and pathological range.

Chromosome	Gene	Dominance	Disease	Location/ direction	Precise coordinate	Number of repeats in T2T/hs1	Normal range	Pathological range
chr19:48,500,000-48,700,000	*DMPK*	AD	DM1	3'UTR/-	chr19:48597740	5CTG	5-37	>50-10000
chr3:129,167,827-129,183,896	*CNBP*	AD	DM2	intron/-	chr3:131917530	(CAGG9)CAGACAGGCAGC(CAGG6)	11-30	>50
chrX:67,544,021-67,730,619	*AR*	XLR	Kennedy disease	coding/+	chrX:65975200	23CAG	9-36	>38-68
chr9:27,546,546-27,573,481	*c9orf72*	AD	ALS	5'UTR intron/-	chr9:27584120	8GGGGCC	3-25	>25
chr6:16,299,112-16,761,460	*ATXN1*	AD	SCA1	coding/-	chr6:16200200	CAG(14Q)CATCAGCATCAG(13Q)	6-38	>39-88
chr12:111,452,214-111,599,315	*ATXN2*	AD	SCA2	coding/-	chr12:111575880	22CAG	13-31	>32-500
chr14:92,058,552-92,106,582	*ATXN3*	AD	SCA3	coding/-	chr14:86300540	24CAG	12-44	>55-887
chr19:13,206,442-13,506,479	*CACNA1A*	AD	SCA6	coding/-	chr19:13333140	13CAG	4-18	>20-33
chr3:63,863,867-64,003,462	*ATXN7*	AD	SCA7	coding/+	chr3:63956320	10CAG	4-33	>37-460
chr6:170,554,369-170,572,859	*TBP*	AD	SCA17	coding/+	chr6:171935500	38CAG(or CAG/CAA)	25-40	>43-66
chr20:2,652,632-2,658,393	*NOP56*	AD	SCA36	intron/+	chr20:2683195	4GGCCTG	5-14	>650-2500
chr8:104,489,236-104,589,258	*LRP12*	AD	ALS2	5'UTR/-	chr8:105716420	12CGG	13-45	>90-130
chrX:147,911,919-147,951,125	*FMR1*	XLR	FRAXA	5'UTR/+	chrX:146176700	20CGG	5-50	>200
chr4:39,287,456-39,366,362	*RFC1*	AR	CANVAS	intron/-	chr4:39318080	12AAAAG	variable	>400AAGGG
chr9:69,035,752-69,079,076	*FXN*	AR	Friedrich's ataxia	intron/+	chr9:81210840	6GAA	5-34	>66-1300
chr13:101,708,804-102,404,443	*FGF14*	AD	SCA27	coding/+	chr13:101377580	50GAA	5-250	>250
chr1:56,994,778-57,424,060	*DAB1*	AD	SCA37	intron/-	chr1:57245940	17ATTTT	7-400(ATTTT)	ATTTC>31-75
chr16:66,427,295-66,482,833	*BEAN1*	AD	SCA31	intron/+	chr16:72284670	13TAAAA	variable	TGGAA>110(+TAGAA)
chr22:45,671,834-45,845,307	*ATXN10*	AD	SCA10	intron/+	chr22:46303900	14ATTCT/ATTGT	10-32	>280
chr5:146,580,742-146,878,757	*PPP2R2B*	AD	SCA12	coding/-	chr5:147414740	15AGC	4-32	>43
chr13:70,099,727-70,157,682	*ATXN8/ATXN8OS*	AD	SCA8	3'UTR/+	chr13:693612448	CAG/CTG_11ACT_15GCT	15-50	>74-250
chr4:3,074,681-3,243,957	*HTT*	AD	HTT	coding/+	chr4:3073630	21CAG	6-35	>36-250
chr12:6922459-6944321	*ATN1*	AD	DRPLA	coding/+	chr12:6947900	19CAG	3-35	48-93
chr16:72,782,885-73,048,128	*ZFHX3*	AD	SCA4	intron/-	chr16:79364620	18CAG	14-26	>42-74
chr21:43,773,950-43,776,308	*CSTB*	AR	Unverricht-Lundborg (ULD)	5'UTR/-	chr21:42132115	6CCCCGCCCCGCCG	2-3	>30
chr16:67,842,320-67,844,195	*THAP11*	AD	SCA	coding/-	chr16:73638607	29CAG	20-38	>45-100
chrX:111,245,099-111,270,483	*CAPN6*	NA	NA	5'UTR/-	chrX:109718596	12CAG	NA	NA
chr4:139,716,753-140,154,184	*MAML3*	NA	NA	/	chr4:30705876	12CAG	NA	NA
chr3:113,648,385-113,696,642	*KIIA2018; USF3*	NA	NA	coding/-	chr3:116378433	14CAG	NA	NA
chr17:57,971,552-57,988,254	*VEZF1*	NA	NA	coding/-	chr17:58853260	CAG	NA	NA

chr13: 35,473,419-35,478,764	*MAB21L1*	NA	NA	intron/-	chr13:34694663	13CAG	NA	NA
chr12:6,666,477-6,689,375	*ZNF384*	NA	NA	coding/-	chr12:6678263	15CAG	NA	NA
chr1:154,697,455-154,870,281	*KCNN3*	NA	NA	coding/-	chr1:154009063	20CAG	NA	NA
chr15:99,565,984-99,716,488	*MEF2A*	NA	NA	coding/+	chr15:97476310	11CAG	NA	NA
chr12:102,957,674-102,960,513	*ASCL1*	NA	NA	coding/+	chr12:102919200	12CAG	NA	NA
chrX:23,944,919-23,951,545	*SUPT20HL1*	NA	NA	coding/+	chrx:23948417	21GCT	NA	NA
chrX:9,306,149-9,594,954	*SHROOM2*	NA	NA	intron/+	chrx:9399120	15CTG	NA	NA
chr9:20,353,454-20,638,229	*MLLT3*	NA	NA	coding/-	chr9:20428160	42AGC	NA	NA

NA not available.

Supplementary Table 3: Hac vs sup basecaller. Comparison between samples basecalled using hac and sup Dorado models. Sup basecaller significantly improves all the metrics ($p < 0.05$, Wilcoxon test).

Sample	Basecaller	Mismatch rate	Insertion rate	Deletion rate	Soft-clip rate	NM per kb	Homopolymer error rate
dm_5	hac	0.01732	0.008572	0.01600	0.07577	41.901	0.03347
	sup	0.01134	0.005306	0.009183	0.05722	25.833	0.02307
dm_16	hac	0.01708	0.008534	0.01484	0.08037	40.448	0.03213
	sup	0.01009	0.004652	0.007718	0.05357	22.460	0.01959
pmm_1	hac	0.006063	0.004061	0.006135	0.03296	16.259	0.01097
	sup	0.005814	0.002985	0.004158	0.02996	12.957	0.01489
dm_6	hac	0.01944	0.009146	0.01731	0.07301	45.900	0.03517
	sup	0.01251	0.005473	0.01004	0.05212	28.023	0.02351
dm_3	hac	0.01721	0.008087	0.01462	0.06855	39.915	0.03050
	sup	0.01017	0.004515	0.007762	0.03936	22.453	0.01936
dm_2	hac	0.01801	0.008565	0.01552	0.06456	42.098	0.03225
	sup	0.01147	0.005049	0.008790	0.04171	25.306	0.02158
cnv_2	hac	0.01660	0.007655	0.01402	0.04772	38.277	0.03020
	sup	0.01298	0.005811	0.01052	0.05665	29.313	0.02808
dm_25	hac	0.01699	0.007910	0.01281	0.16078	37.713	0.03259
	sup	0.01053	0.004255	0.006613	0.14268	21.395	0.01858

Supplementary Table 4: T2T vs hg38 alignment. Comparison between samples mapped to T2T and hg38 genome reference. T2T significantly improves all the metrics ($p < 0.05$, Wilcoxon test).

Sample	Reference	Mismatch rate	Insertion rate	Deletion rate	Soft-clip rate	NM per kb	Homopolymer error rate
dm_30	T2T	0.003894	0.002686	0.003903	0.02844	10.4831	0.01501
	hg38	0.005515	0.003337	0.004394	0.03299	13.246	0.01612
dm_31	T2T	0.005350	0.002971	0.004436	0.03425	12.757	0.01588
	hg38	0.007931	0.003474	0.005282	0.03708	16.687	0.01703
pmm_1	T2T	0.003735	0.002422	0.003515	0.02661	9.672	0.01372
	hg38	0.005814	0.002985	0.004158	0.02996	12.957	0.014890
dm_34	T2T	0.003731	0.002327	0.003412	0.02820	9.469	0.01349
	hg38	0.005873	0.002853	0.004171	0.03119	12.897	0.01456
dm_33	T2T	0.005220	0.003258	0.004893	0.07054	13.371	0.01781
	hg38	0.007655	0.003837	0.005751	0.07362	17.243	0.01899
dm_32	T2T	0.004174	0.002640	0.003787	0.02739	10.600	0.01435
	hg38	0.006520	0.003174	0.004565	0.03073	14.259	0.01545
dm_28	T2T	0.004683	0.002788	0.004012	0.03241	11.482	0.01492
	hg38	0.007112	0.003338	0.004752	0.03537	15.203	0.01599
dm_29	T2T	0.003892	0.002486	0.003390	0.02774	9.768	0.01342
	hg38	0.006037	0.003009	0.004002	0.03142	13.047	0.01443
dm_18	T2T	0.005996	0.003112	0.004897	0.02924	14.005	0.01657
	hg38	0.009064	0.003606	0.005911	0.03134	18.581	0.01751

Supplementary Table 5: Coverage at regions of interest. Mean coverage at each region of interest for each sample sequenced using native ONT. The median coverage is 10x. See Table S5.

Sample	AR	ASCL1	ATN1	ATXN1	ATXN10	ATXN2	ATXN3	ATXN7	ATXN8	BEAN1	C9ORF72	CACNA1A	CAPN6	CNBP	CSTB	DAB1	DMPK	FGF14	FMR1	FXN	HTT	KCNN3	KIIA2018	LRP12	MAB21L1	MAML3	MEF2A	MLLT3	NOP56	PPP2R2B	RFC1	SHROOM2	SUPT20HL1	TBP	THAP11	VEZF1	ZFHX3	ZNF384
cnv_7	0	7.3	3.7	5	5.7	6.1	6.5	9.8	7.4	6.1	6.2	2	1	5.2	0	9.5	3	13.8	1	3.6	1	8	7.4	1.2	4	7.4	7	0.5	7.3	4.9	8.3	0	0	3	0.8	1	5.5	5
dm_5	7.8		0.1	11	13	7.6	8	13.9	12.5	12.9	14.9	18.4		8.7	18	12.1	11.3	0	0	11.9	11.8			25.2					14.2	15.7	22.2			14.4	12.5		11.5	
cnv_5	23.8	22.9	18.5	30.7	15.5	19.3	17.7	16.4	27.1	19.4	26.4	10.8	24.2	13.9	10.4	27.9	14.8	20.5	27.8	29.9	17.1	16.3	22.1	21.9	30.3	30.7	17	5.1	14.9	27.4	19.1	0	0	19.7	4.4	20.3	22	20.8
dm_13	7		6.8	5	5	6	3	3	6.8	6	3.2	4		5	6	4.5	3.1	3.5	0	5.2	5			5					0.5	6.5	9.3			3.1	4.9		3	
sc_9	20.7		14.8	20.1	11.6	16.2	12.6	25.3	21.1	17.2	22.7	15.1		19.9	6.9	19.9	15.9	19.3	17.2	21.8	18.9			18.9					10.9	29.9	17.8			18.5	7.3		21.5	
dm_14	10.2		7	11.8	6	15.5	14.8	16.2	11.4	8.9	7.8	9.5		9.2	6	15.3	10.5	9.9	0.9	13	14.7			9					7	10	12.2			10.6	14.1		9	
dm_34	9.8	15	7.7	14.6	8.9	6.2	13.7	11.2	0.3	6.8	4.9	8.9	19	13.6	0.6	8.9	7.8	8.6	4.8	1.9	11.4	5.8	9.9	8.7	5.1	0.4	11	0.7	13.2	7.5	5.4	0	0	10.7	1.5	11.2	10.8	10.6
cnv_1	5.2		12.3	17.1	14.6	11.3	14.3	17.7	26.2	17.6	13.6	18.2		16.9	17.8	22.6	13.6	19.3	8.6	19.1	11.7			15.1					14	21.1	11			16.2	14.5		19.1	
dm_12	1.7		2	1	3	5	8	3.5	5	3	0	4.4		6	5	3	3.7	2	0	5.6	7.2			5					7	10	8			10	5		7	
sc_6	23		32	33	28.8	29.6	38.4	24	27.3	23.6	32.8	29.2		30.7	29.1	25.1	36	20.6	0.9	26.2	32			39					28	36	23.7			33.2	25		30.7	
dm_16	10		8.9	4.6	10.8	8	5	5.7	10.7	13	10	14.4		7.3	15.3	10.3	9.7	12.7	0.7	6.3	3.1			12.2					7.2	10	12.8			6	11		10.4	
sc_4	4		1.2	14.5	10	8	4.1	11.3	12	14.6	11	4.5		7.3	10.8	6.8	8.6	1	0	16.5	11			21.8					11.7	12.1	11.6			6.5	6		11.5	
als_5	4.6	6	3.9	2.8	5	1.2	6.4	3.7	2.4	6	4.5	4.3	2	6.1	0.6	5.9	6.4	8.7	1	1.8	1	4.1	8.5	6	6.4	2.4	4	1.5	7.4	9	7.6	0	0	5.4	1.6	3.1	6	7.7
dm_22	6		7.2	6.4	5.8	5	5.2	7.2	6.1	8.9	11.6	10.2		11.1	12.2	9.2	5.6	4.5	9	15	6			10.4					4	10.7	7.4			8.1	6		4.7	
als_3	10.4		2	6.1	7.8	4	3.8	12.3	11	4.3	11.7	6.8		8.2	10.7	5.2	6	0.3	1.3	8	3			12.3					9.3	7.4	2.9			3.6	5.1		2.6	
cnv_8	15.9	31.4	22.8	32.7	30.6	21.8	25.6	29.8	31.1	29.9	23.9	33.3	7	23.4	33	42.2	15.4	26.8	19.4	33.1	24.9	18.1	33.8	38.8	40.2	32.4	35	4.8	35.9	36.6	22.4	0	0	22.8	3.5	25.2	35.1	25.6
dm_6	4.9		0.7	5	4	2	8	4	4.6	7	4	6.1		5	2.9	7.8	4	0.7	0	8.8	8			6					5.6	5	4			4	9		3.8	
sc_7	5.2		12.3	17.1	14.6	11.3	14.3	17.7	26.2	17.6	12.6	18.2		16.9	17.8	22.7	13.6	19.3	8.6	19.1	11.7			15.1					14	22.2	11.1			16.8	14.5		19.1	
sc_5	8.8		5.6	7.9	19.6	11	14.1	14	13.4	11.7	10.6	14.4		16.8	14	14	10	19.5	1	16.8	5.2			12.5					13.2	17.2	8.9			15.4	10.2		13.6	
cnv_4	5.1	4	4.8	11	11.5	4.8	10.1	6.6	3.7	7.7	5.2	7.8	11.5	11.5	0.9	10.8	9.2	10.6	13.3	3.8	6	6.8	11.1	6	8.9	2.8	9.4	1.3	7.8	7.1	8.4	0	0	8.6	2.4	9.4	14	15.6
dm_23	9.8		8.5	14	7.2	8.3	5	7.9	18.6	8.7	11.1	6.2		9.1	6.9	20.3	11.1	11.9	14	19	7.6			15.5					7	10.9	19.1			16.8	11.9		14.8	
dm_9	8.8		1.1	9.6	4	6	13.4	9.6	5	8	5.7	9.2		11.9	0	5.9	6.2	2.5	0	7	0			8					6.2	4	8			6.6	3.7		0	
dm_4	11		0.6	15.3	11.3	20	9	7.1	25.9	12.5	9.7	10		13.4	8.9	8.3	2.4	2.2	0.2	10	11.5			13					10.3	12.9	16.3			8	16.3		14.9	
dm_28	1.2	1.5	7	3.3	5.4	3.5	6	1.2	0.6	9.6	6	3.9	2	3.3	2.2	3	5	9.5	1	1.2	7.8	5.4	4.4	7.3	6.1	1.1	2	0	3	5.9	3.7	0	0	4.1	0.5	3.2	6.3	5.2
dm_2	6		0	5	14.6	8.8	7.6	8	4.1	7.9	4.3	2		16.1	6.3	11	10.4	1.1	0	6.9	9			14					10.5	10.4	6.1			7	2.7		5	
sc_8	16.3		14	19.5	19.7	13.8	12.2	18.7	16.8	13.7	16.3	17		21.6	16.9	17.4	9.3	15.6	16.4	14.6	13.9			17.4					22.6	19.4	22.1			15.3	11.8		17.6	
dm_17	36.4		25.7	26.5	31.3	25.1	26.7	31.3	37.8	20.6	28.4	30		24.2	22.4	39.4	22.4	28.6	1.1	28.7	25.3			32.8					27.1	32.4	33.2			25.3	21.4		26.6	
als_9	5	7.9	2.4	4.4	5.7	3	7.1	4.2	0.9	3	4.4	4.1	6.3	7.5	0.1	4	3.6	2.2	1	0.3	6	3	8.7	7	8.1	1.1	4.3	0	1.4	4	6.1	0	0	6.9	0.3	6	4	4.8
sc_12	8		5	15.2	4.8	5	7.7	8	11.1	6	12	8.9		10	4	9.6	5	9	0	11.2	4			8					7.2	11	5.1			3	11		5	
dm_10	5		0.6	5	6.6	1.2	1.2	4.4	1	4	3.9	1		2	1	0	2.4	0	0	2.4	2			7.3					5.4	3.2	1			5.3	4		2	
dm_21	1		8.8	6.6	2	1	5.6	6.2	6	1	4	1		2	3	10.4	5	1	1	4	2.5			1					1	6.3	2			5.1	3		4	
ne_8	10.1		0.5	10.1	19.6	7.7	17.7	14.2	7.1	10.6	11	10		15.6	12.5	19.7	13	2.7	0.7	14.2	21.2			9					9	20.2	26			4.9	12.2		19.2	
dm_32	2	5.4	1	7.8	4.6	1	6.8	7.9	2.4	7	2.7	4	3	4.2	1.3	4.2	3	3.1	5.4	1	4	1.2	1	4.6	8.6	0	4	0	5	5.9	9.7	0	0	8.6	1.4	6	4.1	5.5
dm_1	5.4		0	12	10.5	10.5	14.3	10.4	16	20.7	11.4	10.6		11	1.1	10.7	12	3.3	0.1	12.9	1.9			8					15	9.1	12.4			7	0.5		1	
cnv_2	2		4	7.9	5.7	6	4	3.9	8.4	8.9	5	8		9.8	3.5	9.6	2.1	11.4	4.1	11.4	3			8.9					9.1	8.4	5			6.6	4.8		11.4	
cnv_3	2	12	6.1	5.8	0	7.3	1.2	8.4	6.5	0.8	5	6.2	0.5	1.6	5	5.3	1.2	11	0	0.6	6.9	1.4	0.9	0.4	1.1	1.3	2.6	1.1	8.2	1	11.1	0	0	0	9.1	1.3	2.3	21.2
ne_12	7.1		1	4.2	10.2	7	3	6	6	4	5.5	10.7		5.7	5	6	7	0.4	0.2	8.5	3.9			4.1					8.3	8.8	7.4			8.2	2		6.6	
ne_10	5.9		11.8	10.9	6.5	8.2	7.1	5	4.5	9.6	6.6	4.8		9.3	5.3	9	6.5	3.1	0.7	6.7	8.8			3.3					3.4	6.2	6.9			8.9	4.9		2.6	
dm_30	11.6	6.5	5	10.5	6.9	9	6.7	6.3	0.7	13.3	7.5	8	7.6	7.7	0.4	11	9	5	11.9	0.8	8.6	6	7.7	9	8	0.5	10	0.7	5.4	5.5	7	0	0	10.6	1.2	6	9	6
dm_26	10.7		5	11.3	7.5	6.3	6.6	5	10.2	5.5	4.8	9.2		6	7.1	6.2	8.1	4.1	6	11.1	6.1			8.3					5.8	8.9	2			7	6		8	
dm_18	15.3		14.7	28.3	26.2	24.3	23.3	26.5	29.4	13.7	36.6	15.4		23.2	18.7	22.2	22.2	21.2	1	26.9	27.3			25.5					17.7	20.3	21.9			15.5	23.4		14.8	
cnv_6	4.6	13.8	5.7	12	8.6	9.4	6.6	12.1	1.7	9.1	15.5	5.4	5.5	9.6	1.6	15.4	9.2	9.8	3.4	1.4	10	5.2	17.6	17.6	16.4	3.3	14.7	2.1	4.2	13.6	8.1	0	0	4.5	1.6	8.8	12.3	16.3
ne_3	19		25	25.5	18.8	23.3	39	28.5	29	34	25.5	30.8		21.2	27.6	31.5	19	16.9	0.6	14.4	35.5			34.8					29.7	29.7	24.1			25.4	28.4		30.3	
ne_17	6.8	7	6.7	8.9	9.8	9	13.8	10.6	8.6	5.4	3.3	5.7	5.1	12.5	8.7	14.3	17	13.9	4.3	10	12.4	8.9	10.5	11.4	11.4	14.7	13.5	1.3	13.4	8.3	8.7	0	0	10.2	1.2	11	11.7	10.2
dm_24	0		1	6.1	4.6	1.1	4.7	3.2	3.9	3.9	0	1		1	2.4	6.5	1	6.1	1	5	0								4.8	2.4	1.8			0	1.3		4	
als_8	11.9		3.8	11	8.4	8.8	14.6	9.3	18.2	8.4	3	6.5		7.4	9	12	9	8.8	0.7	7.8	7.7			10.4					2	13.4	14			7.5	8.4		12	
als_4	3		6.8	3.7	4	2	7	8.4	6	10.1	8.9	4.4		8.5	11.1	3.5	4	5	0	4	6.9								10.7	5	4			3	5.4		5	
ne_1	0		0	1	5.8	2	7	0	7.2	2.6	6	3.8		1	4.5	2	6	0	0	1	4			3					1.3	3	0.1			3	1		3.3	
ne_5	1		2.8	3.8	2.1	3	1	2.6	1	3	1.9	[illegible]		1	2	4	2	0	0	3.3	4			2					0	4	0			4	2		4	
dm_25	0		2.3	1.8	4.9	4.1	4	2.9	3.3	3.6	5.5	1.8		6.6	1.7	1.1	2.3	4.8	1.5	2.5	1.3			1.5					3.7	1	6.6			4.2	0.9		0.6	
hd_1	3		0.1	1	3.4	1.1	10.5	3	5	6	3	3.6		2	3	3	1	0	0	7	4			4					4	0	0			4.7	5		3	
dm_8	12.8		0.9	6.6	7.7	10	13.8	14	17.5	7.8	14.3	12.9		9.2	1.2	10.3	10.9	1	2.3	10.1	1			23.7					17.8	5	16			17	2.6		0.7	
ne_4	6		4.6	13.4	15.9	14.6	7.2	10.4	12.7	7	10.8	6.2		14	7.6	8.9	9	4.2	0.5	9.1	8			13.3					7.6	9	20.6			7	10.2		14.1	
ne_11	3		1	4.5	12	7	9.5	4	12	9.8	8.1	8.9		7	2.1	6	8.9	0.7	0.8	3	6.2			10					13.7	4.8	11			6.5	15.5		8.8	
dm_3	17.5		0.8	11	13.8	7.7	12.4	6.6	8.5	15	8.4	7		9.6	10.3	11.1	7.7	1	0.4	3.7	8.3			17					8.7	12.4	7.8			16.6	12.2		13.5	
dm_27	4		9.6	5.4	3.6	6	7	10.8	6.6	11.8	11.8	3		8.5	7.5	12.4	5.4	7.9	6	4.6	12			12					11	7.3	10.9			6	9.3		6	
dm_20	6.6		14.9	18.2	17.5	20.5	20.8	12.4	20	19.9	19.7	12.8		17.4	17.5	21.2	18.8	15.9	10.6	10.2	20.2			18					8.3	26	14.3			14	18.6		16.2	
dm_19	14.2		16.3	11.9	18.1	20.8	8.9	20.7	29.8	24.1	13.5	15		16.4	18.3	20.7	13	16.6	0	23.5	16			17.5					16.2	12.1	20.1			20.5	18		24.9	
als_6	0	6.2	5.4	4.6	6.5	4.4	6.1	8	3.2	5.5	5.1	6.6	3.6	8.6	1.5	11.6	5.8	9.4	7	1.8	10.1	8	2.5	10.7	8.4	0.7	6.4	1.8	10	6.3	9.2	0	0	6.2	0.9	8.4	11.1	8.4
sc_2	9.7		1.9	7.3	5.6	8	10.5	11	7	10	7.5	8.7		5.2	12.3	12.3	9.4	1.7	0.7	4	4.2			12.9					7.4	14.1	7.2			11	5.5		12.5	
sc_11	27.8		21.5	18.5	15.9	25.3	19.8	21.4	23.4	18.3	21.7	18.9		13	20.9	33	26.4	20.4	2.1	33	39			19.5					24.7	16.8	12.8			18.5	18.5		11.4	
cnv_8	4.5	6	3.4	10.5	5.2	3	3.2	5	7.8	5.1	7.1	5.5	1	4	5.9	4.9	2	10.4	3	3.6	2.1	3	3.6	10	3.8	3.4	5	1.5	5	6	2.6	0	0	4.3	0	5.5	6.6	4
ne_9	1		4	7	2	6.4	2.5	8.8	4.6	5.1	3.2	1.4		5	7.4	5	5.9	6.6	0	10.5	4.4			6					6.6	4.4	3.6			2.9	6.6		4.8	
dm_15	10		7	4.8	7.2	10.4	4.8	6	2.4	5.9	4.8	5.4		3.5	2	14.8	5.8	13.1	1.3	6	8			8.6					6.1	9.7	8.3			8.6	5.2		12	
ne_2	25.5		15.4	30.2	17.2	10.7	25.9	17	17.1	27	25.9	14.1		17.5	14.3	17.3	15.2	19.7	3	13	19.4			21.9					13.2	26	26.5			22	23.1		17.7	
ne_16	11.1		10	11.5	14.8	15.8	19.5	12.7	19.9	6.9	10.6	10		11.2	17.7	11.3	12.7	13.8	7.2	9	12			8.4					13.7	12.5	6.8			12.7	9.2		15	
sc_10	12		9.5	9.8	16.6	16.8	14	17.2	20.4	9.1	15.7	18.2		17.3	16	11.8	16.3	10.2	0.3	18.7	8.4			20.9					18.4	15.1	14.1			11.8	9.7		8.1	
ne_14	5.1		10.3	8.2	12.7	9.1	10.9	11.5	15.2	9	11.7	12.5		10.5	8.1	13.7	9.5	8.3	7.3	9.5	7.8			13.6					9.2	14.9	13			10.8	8.5		11.2	
sc_3	1.1		0.2	0.5	0.4	0.1	0.2	0	0.5	4.8	1	0.8		2	0	0.8	1	0.6	0	1.3	0			2.3					1.3	1.1	1			0.9	1.6		1.7	
als_2	2		2.9	5.6	1.9	6	3	3.2	3	6.7	6.3	7		3	4	6.5	4.5	6.2	0	5.4	7.5			0					2	4	4			4.8	7.9		3.2	
sc_14	7		0.1	11	12.6	5	9.2	11.8	5.6	11.4	14	11.1		8.4	13.3	15.5	6	1.5	1.1	16	10			16.1					7.6	16.3	6.8			12.2	9		6.1	
dm_11	2.2		4	4	6	2	7.4	8.4	6.8	1.7	6	3		4.2	7	6.8	2.4	1	1	7	3			1					5.4	6.2	2.1			5.3	2.1		6.4	
dm_7	15.8		0.9	9.6	10	9.6	9.1	11.2	10.3	4	11	5		6.4	0	11	5.9	1.7	1.6	12.1	6.8			11					9.4	12	5.9			9.5	10.9		4	
als_1	14		9	13.9	5.8	8	15.4	8.7	5.1	7.6	5.2	3.4		6.3	9.7	10	9	1.6	1.2	13	10.3			16.1					5.6	9	5.5			16.2	4.7		12.3	
kd_1	3	4.1	5	5.4	7.6	8.2	3	8.7	0.6	3.1	2.3	4.8	2	6	0.7	2.1	5.5	8.8	1	0.8	8	7.2	0.3	12.7	6.3	1.5	5.9	1.4	10.4	7.1	10.2	0	0	5.1	0.2	5.5	1	3
pmm_1	2.4	5.7	10	8.1	4.1	10	6.2	9	1.7	5.1	8	2.6	4.4	2.3	0	6	9.3	3	1	0.2	4	3.3	7.6	7	8	0	7.4	1.2	7.4	8.7	4.7	0	0	7.8	0	3.7	10.1	2.6
als_7	6.3	15.2	6.5	12	5.5	8.8	9.1	8.5	1.5	8.6	6	8.9	13	8.1	0	8.9	10.9	16.5	12.8	3.1	15.3	5	14.8	10.7	9.8	1.2	11.3	0.7	11.5	7.9	9.6	0	0	14.8	0.5	4.6	1.9	8.6
ne_15	4		0.8	8	5.3	3	6.4	10	6.6	4	8.9	5		4.2	9.3	8.4	1	0.8	0.6	5.5	15			22.4					3.9	6	6.2			15	11.8		5.5	
sc_13	5		0.8	17.5	15.4	12.4	7	10.3	13.6	15.1	9.3	7.9		9.9	10	9.2	2.8	0.1	0	10.9	8			13					3.6	8.3	7.3			9.8	4.4		3.7	
dm_31	12.3	4.8	5.1	8.2	7.7	4.1	3.1	5.6	1	0.2	4.7	4.2	9.3	6.1	0.6	7.8	3.3	11.1	5.8	1.2	12.4	6.5	8.6	5	5.8	0.9	6.1	1.9	6.7	2.8	4.1	0	0	8.3	2.8	11.2	5.1	4.7
dm_33	1	6.2	4	8.3	4	6.1	3.9	6.5	1.5	7	11.7	11	2	6.1	0.6	7.8	5.4	8.8	2.9	0.2	3.6	5.7	8.6	8.5	9.9	0.6	5.8	0.8	13.2	10.7	4.7	0	0	5.8	0.8	6	4.1	9.4
dm_29	4	3.4	1.9	6.2	6	2.7	2.3	7	0.9	3	2.2	0	3.4	0.2	0	6.7	7.7	5.4	3.7	1.3	0.8	5.8	6.8	3.2	5.7	0.2	4.5	0	3.3	7	9.5	0	0	2.8	0	1.9	7.4	3.5
sc_1	8.3		3.8	12.4	9.7	16.2	13.3	10	14.2	8.5	19	13.8		19.8	13.7	12.5	12.4	3.4	1.4	21.6	4			22.4					8.6	13.7	9.6			15.3	13		14	
ne_13	12.3		12	22	8	16.4	14.9	15.3	18.1	14	9	13		10.5	10.2	15.8	9	28.1	0	15.9	6.6			15.3					8.7	15	6			9.3	12.7		11	
als_10	0	1	6	7	0	7	0.9	9	8.9	0.6	11.5	4.6	0.4	0.6	6	7.8	1.3	2.7	1.3	1	5.1	0	1.5	1.5	0.6	1.2	0.7	0.7	9	1.9	7	0	0	0	4	2.7	0.2	2.9
als_11	3	8.5	6	5.2	7.9	3.2	6	7.7	6.5	7	5	2	1.6	7.5	10.9	11.8	4.3	9	7.9	8.7	7.9	2	8.7	13.5	7.6	9	9.4	1.2	7.1	7.2	12.7	0	0	10.5	2.7	11.7	10.3	6.4
fxn_1	4.3	16.3	11.1	8.8	9.4	10.4	10.5	9.5	19.2	12.8	16.7	11.7	7.5	15.2	22.3	8.2	12.6	13.2	7.5	6.8	7.8	12.1	13.6	9.9	13.8	15.5	15.7	5.2	10.3	20.3	20.7	0	0	16.1	5.3	8.3	11.9	8.1
ne_18	14.9	37.1	32	40.2	23.5	29.3	30.2	44	33.1	34.1	30.3	37.6	17.4	30.3	40	39.5	23.5	24.3	14.7	35.4	29.7	21.2	23.1	35.8	28	32.6	37.3	4.9	34.9	36.5	26.2	0	0	28.2	11.1	22.4	27.6	35.2
ne_19	17.4	19.8	13.2	12.3	17.1	18.4	8.3	18.6	17.1	12.6	9.9	13.2	14.5	12.9	13.4	13	15.4	10	15.5	19.4	16.3	8.1	20.4	18.2	10.8	16.1	14.8	1.9	12.6	26.8	13.1	0	0	15.4	2.3	11.2	13	11.7
ne_20	17.8	17.5	18.4	13.5	17.5	10	10.8	17.7	16.4	15.4	11	8.1	12	10.2	7.8	15.3	11	10.5	4.9	18.4	21.8	10.7	24.4	12	20.2	16.4	23.9	2.6	6	19.1	17.7	0	0	17.1	3	11.8	22.2	11.1
ne_21	3.6	10.3	9.3	7.1	8.6	7.6	8.6	6.3	9.8	6	9.7	8.6	3.3	10.5	4.4	5	[illegible]	10.5	1.3	1.5	8.6	4.9	11.1	9.2	3.3	7.8	5.4	1.3	8.7	8.7	6.9	0	0	6	1.7	5.1	3.4	4.8
ne_22	11.6	10.5	6.8	14.7	11.5	19.7	10.4	8.9	14.7	10.6	10.1	15	16.9	9.9	8	16	10.6	12.3	15	14.2	17.7	12.8	11	11	12.8	11.7	6.8	2.2	5.4	19.4	12.1	0	0	9.5	2.1	10.7	6.1	10.7
ne_23	14.9	15.1	11	21	20.3	9	14.8	12.7	9.5	7.4	14.4	8.1	14.7	19.6	6.1	17.7	11.5	15.7	14	8.4	10.1	11.7	18	15.7	13.6	11.4	14.1	3.9	13.2	16.7	13.5	0	0	15.1	4.3	15.3	13.5	8.8
ne_24	9.6	12.3	6.5	7.9	12.4	11	8.2	7.3	11.8	13.5	14.6	9.4	17.7	10.4	5.1	9	17.2	11	8.2	6.6	9.2	12.9	10.1	10.7	18.4	17	6.9	4	13.3	12.3	13.5	0	0	10	3.9	13.4	14.9	16.6
ne_25	11.5	18	16.6	11.5	12.3	12.3	14.3	15	20.7	11.7	14.4	11	7.9	15.4	15	19.7	6.7	17.5	10.8	17	12.8	11.6	19.4	19.7	21.7	17.2	18.7	2.7	16.7	10.8	15	0	0	12.9	2.4	14.2	23.6	11.7
ne_26	7	13.3	7.7	10.4	19.2	13	20.3	12.1	10.8	13.9	12.2	13.3	5.1	14.2	15.5	25.4	8.5	15.3	6.1	16.5	12.3	11.1	18.1	19.6	15.5	12.2	16.2	2.6	15.3	18.8	10.1	0	0	14.7	1.7	18.7	15.8	14
ne_27	14	14.9	15.2	16	12.7	20	13.5	17.1	9.9	13	20.1	13.4	15.5	17.7	16.7	20	11.1	14.5	19.7	20.7	9.7	23.2	18.5	15.6	15.1	20	16.5	4.2	16.8	21.1	13.7	0	0	16	5	20.9	21.2	18
ne_28	15.2	22.9	13.8	21.1	23.2	18	24.9	20.7	42.3	20.9	26.2	24.5	17.9	26	20.2	16.8	15.7	30	15	17.6	19.2	15.4	29.1	28.3	24.7	31	33	2.2	21	29.5	23.4	0	0	29.5	5.1	17.7	24.9	9.9
sc_14	42.1	49.7	24.7	43	40.4	21	44.4	42.7	36.7	39.2	26.1	27.8	37.3	33.2	37.9	33.8	33.5	38.4	44.9	43	38.1	38.9	40	31.3	48.9	46.5	49.7	5	30.5	45.3	40.1	0	0	44.7	9.3	43.2	32.8	33.2
sc_15	19.9	28.6	23.4	22.8	28.9	13	26.2	29.4	31.8	21.5	26.9	14.6	21.5	20.1	31.2	20.9	19.9	25.6	30.7	23.4	24.6	16	29.6	25.2	24	19.5	23.6	5.7	18.6	17.5	18.5	0	0	19.2	6.1	23.6	29.2	27.6
sc_16	3.4	11.7	16.6	8.1	13.1	6	11.7	8.2	10.2	12.2	13.3	7.2	4	10.7	10.1	10.2	11.6	9.4	7.8	8.4	6.6	12	17.6	8	10.5	9.4	17.9	4.8	2.6	7.7	11	0	0	8.8	1.8	7.8	10.8	17.1
fxn_2	11	12.1	4.8	6.6	8.8	9	7.1	10	3	10	4.9	5.3	13.2	4	0.9	11.2	5.4	11.7	8	0.6	5.1	8	6.5	5.4	9.1	0.7	12.7	0.8	11.1	9.6	6.4	0	0	6.1	1.1	9.6	6	10.6
cnv_9	23.7	20.2	6.7	10.6	9.8	11.1	6.9	11.4	18.6	18.6	9.7	6.4	16.7	12.7	7.9	14	3.4	20.5	14.8	14.1	12.1	12	22.3	16.4	16.4	20.6	18.3	2.9	14.5	20.2	14.2	0	0	14.3	3.7	15.7	19.8	11.1
cnv_10	7.8	15.1	21.6	18.9	27.1	19.7	28.3	14.4	17.8	13.8	14.2	15.9	10.7	25.3	10.5	23	9.1	12.7	12.8	14.6	13.2	22.8	20.6	14.3	18.3	21	23.7	4.1	23.2	19.4	22.1	0	0	24.4	7.1	23.8	23.3	16.7

Supplementary Table 6: AS effectiveness. Enrichment ratio, Fold enrichment, and number of on-target bases obtained using AS for the cohort of patients with REDs. Some patients show very small FE and ER.

Run	Enrichment ratio	Fold-enrichment	On-target bases (Gb)	Run	Enrichment ratio	Fold-enrichment	On-target bases (Gb)
dm_9	7.28	6.59	0.0286	ne_9	4.18	3.78	0.0180
dm_10	7.23	6.54	0.0152	ne_12	5.67	5.13	0.0263
dm_5	8.09	7.33	0.0501	ne_10	4.12	3.73	0.0283
dm_20	5.65	5.11	0.0584	ne_16	8.05	7.28	0.0484
dm_23	5.63	5.09	0.0418	sc_1	5.68	5.13	0.0512
dm_17	13.63	12.33	0.1054	ne_1	9.08	8.21	0.0147
dm_16	7.89	7.15	0.0348	ne_13	7.06	6.39	0.0521
dm_19	8.83	7.99	0.0725	ne_3	20.51	18.56	0.111
dm_1	6.49	5.87	0.0409	ne_14	7.30	6.61	0.0433
dm_14	6.14	5.56	0.0388	ne_2	6.16	5.57	0.0707
dm_11	6.60	5.97	0.0175	sc_10	5.93	5.36	0.0515
dm_21	12.26	11.09	0.0170	sc_11	13.29	12.02	0.0757
dm_12	10.18	9.21	0.0208	sc_14	4.459	4.03	0.0398
dm_13	7.78	7.04	0.0211	cnv_2	5.19	4.69	0.0267
dm_22	8.59	7.78	0.0338	hd_1	5.80	5.25	0.0151
dm_6	6.67	6.04	0.0257	als_8	4.78	4.33	0.0363
dm_3	6.90	6.25	0.0421	als_1	5.06	4.57	0.0398
dm_2	8.48	7.67	0.0362	als_3	4.69	4.25	0.0251
dm_15	6.46	5.85	0.0309	als_4	5.01	4.54	0.0271
dm_18	11.32	10.24	0.0849	als_2	5.47	4.95	0.0171
dm_24	4.31	3.89	0.0102	als_10	5.87	5.31	0.0313
dm_8	6.99	6.32	0.0472	dm_30	4.22	3.79	0.0254
dm_7	9.56	8.65	0.0413	dm_31	2.04	1.83	0.0222
dm_4	8.24	7.45	0.0488	pmm_1	3.27	2.94	0.0202
dm_27	4.94	4.47	0.0289	dm_34	2.76	2.48	0.0270
dm_25	1.32	1.19	0.0196	dm_33	2.71	2.43	0.0188
dm_26	7.27	6.58	0.0316	dm_32	2.69	2.43	0.0156
sc_3	0.806	0.729	0.00516	dm_28	2.36	2.12	0.0167
sc_4	5.04	4.56	0.0439	dm_29	3.05	2.75	0.0158
sc_5	3.76	3.39	0.0497	ne_17	2.89	2.59	0.0336
sc_12	6.67	6.03	0.0353	cnv_4	2.32	2.09	0.0286
sc_6	7.68	6.95	0.1226	cnv_3	6.66	5.99	0.0465
sc_2	5.46	4.94	0.0369	cnv_8	3.05	2.75	0.0174
sc_9	7.83	7.09	0.0683	cnv_5	2.79	2.52	0.0241
sc_8	8.09	7.32	0.0693	cnv_6	2.67	2.40	0.0304
sc_7	6.67	6.03	0.0577	cnv_7	2.77	2.49	0.0189
ne_4	3.79	3.44	0.0375	kn_1	2.29	2.06	0.0185
ne_5	3.95	3.58	0.0115	als_7	2.25	2.02	0.0292
sc_13	5.82	5.26	0.0360	als_6	2.38	2.14	0.0249
ne_11	6.15	5.56	0.0312	als_9	2.32	2.09	0.0161
ne_15	5.78	5.23	0.0283	als_5	2.26	2.04	0.0177
ne_8	6.09	5.51	0.0517	als_11	2.06	1.85	0.0301
fxn_1	1.93	1.73	0.0294	ne_18	4.02	3.75	0.110
ne_19	2.51	2.34	0.0478	ne_20	3.13	2.92	0.0498
ne_21	3.14	2.93	0.0249	ne_22	3.13	2.92	0.0393
ne_23	2.49	2.32	0.0483	ne_24	2.62	2.45	0.0407
ne_25	2.86	2.67	0.0551	ne_26	2.86	2.67	0.0518
ne_27	2.85	2.66	0.0579	ne_28	2.82	2.63	0.0852
sc_14	2.82	2.63	0.129	sc_15	2.63	2.45	0.0767
sc_16	2.81	2.62	0.0383	fxn_2	3.29	3.07	0.0216
cnv_9	3.05	2.84	0.0551	cnv_10	2.42	2.26	0.0732

Supplementary Table 7: Diagnosis prospect of samples with DM1. Diagnostic prospect of samples affected by DM1. Expansion patterns, obtained with NanoExpansion, IGV visual inspection for native and long PCR ONT, genome reference, and SB class are reported. Expansion sizes vary across the same patients, indicating the presence of mosaicism. The agreement between ONT (both native and PCR) and SB genomic classification is optimal.

Patient ID	NanoExpansion	IGV (native)	IGV (PCR)	Reference	Class (SB)
dm_1	(CTG)43(CTC)694(CTG)56; (CTG)1119<br>(CTG)1005; (CTG)727	796; 1119; 1002; 745	296-618	hg38	E2
dm_2	(CTG)1094; (CTG)38(CTC)909; (CTC)857(TG)31(GTG)4(CAC)2(CTG)41<br>(CTG)50(CTC)428	1091; 948	268-776	hg38	E2
dm_3	(CTG)83; (CTG)75 (CTG)75; (CTG)77; (CTG)75	83; 74; 73; 77; 75	71-130	hg38	E1
dm_4	(CTG)1100	1096	246-670	hg38	E3
dm_5	(CTG)137; (CTG)127; (CTG)123	134; 125; 122	104-148	hg38	E2
dm_6	(CTG)11(CTC)413(CTG)8(CCG)(CTG)8(CCG)(CTG)6(CCG)3(CTG)6(CCG)2(CTG)8(CCG)(CTG)58	526	302-803	hg38	E2
dm_7	(CTG)466; (CTG)620	542	86-157	hg38	E1
dm_8	(CTG)146(CTC)344(CTG)2(CCT)4(CTG)144<br>((CTG)79(CTC)688(CTG)50<br>(CTG)59(CTC)610(CTG)1	641; 817; 674	250-822	hg38	E2
dm_9	(CTG)1241; (CTG)16(CTC)1388(CTG)46<br>(CTG)899(CTC)1(CTG)441<br>(CTG)83(CTC)936(CTG)38	1241; 1408; 1341; 1257; 1056	295-1071	hg38	E3
dm_10	(CTG)115(CCG)41(CTG)53	209	196-321	hg38	E2
dm_11	(CTC)1198(CTG)28; (CTG)799	1233; 797	169-538	hg38	E2
dm_12	(CTG)131; (CTG)204	131; 204	124-226	hg38	E2
dm_13	(CTG)315	315	243-712	hg38	E2
dm_14	(CTG)383; (CTG)672; (CTG)1053<br>(CTG)30(CTC)831(CTG)12;	383; 672; 882; 1050	211-660	hg38	E2
dm_15	(CTG)25(CTC)948; (CTG)818;<br>(CTG)35(CTC)721(CTG)60	976; 816; 812	154-990	hg38	E2
dm_16	(CTG)199; (CTG)165; (CTG)159	199; 165; 159	158-412	hg38	E1
dm_17	(CTG)117; (CTG)10(CTC)53(CTG)33; (CTG)34(CTC)39(CTG)56; (CTG)110; (CTG)98; (CTG)134; (CTG)93; (CTG)94; (CTG)94; (CTG)94	115; 98; 127; 109; 95; 114; 91; 94 (3)	83-146	hg38	E1
dm_18	(CTG)47(CTC)337(CTG)86<br>(CTG)41(CTC)477(CTG)30<br>(CTG)66(CTC)496(CTG)10;<br>(CTG)1117<br>(CTG)46(CTC)510(CTG)42<br>(CTG)60(CTC)710(CTG)91(CTC)163(CTG)73<br>(CTG)71(CTC)648(CTG)5; (CTG)727	470; 550; 574; 611; 1098; 1117	182-719	hg38	E2
dm_19	(CTG)1404; (CTG)1002; (CTG)888; (CTG)596; (CTG)30(CTC)887(CTG)47;<br>(CTG)77(CTC)924(CTG)93	1400; 1001; 887; 594; 968; 1094	184-731	hg38	E2
dm_20	(CTG)28(CTC)845(A)38(CTC)67(CTG)38	990; 1275	274-1134	hg38	E3

	(CTG)52(CTC)1223				
dm_21	(CTG)92; (CTG)99	92; 99	88-118	hg38	E1
dm_22	(CTG)42(CTC)716(CTG)40; (CTG)27(CTC)902(CTG)4	798; 933	242-846	hg38	E2
dm_23	(CTG)47(CTC)6(CTG)4(CTC)277(CTG)52; (CTC)118(CTG)74	386; 191	123-155	hg38	E1
dm_24	(CTG)45(CTC)600(CTG)45(TG)	690	205-846	hg38	E2
dm_25	(CTG)129	129	/	hg38	E1
dm_26	(CTG)35(CTC)253(CTG)36; (CTG)61(CTC)152(CTG)297; (CTG)558	558; 510; 324	292-574	hg38	E2
dm_27	(CTG)107(CTC)82(CTG)167(CTC)149(CTG)147; (CTG)107; (CTG)70(CTC)4(CTG)4(CTC)85(CTG)34; (CTG)246	540; 246; 197; 106	69-137	hg38	E1
dm_28	(CTG)307	307	250-404	T2T	E2
dm_29	(CTG)77(CTC)2166(CTG)13; (CTG)30(CTC)974(CTG)53; (CTG)36(CTC)788(CTG)4; (CTG)59(CTC)700(CTG)52; (CTG)15(CTC)446(CTG)27	2255; 1057; 828; 811; 488	211-668	T2T	E2
dm_30	(CTG)151; (CTC)69(CTG)51; (CTG)109; (CTG)91	151; 120; 109; 91	96-141	T2T	E1
dm_31	(CTG)514	513	132-463	T2T	E2
dm_32	(CTG)4(AAA)66((TTG)2(CTG)33	147	125-149	T2T	E1
dm_33	(CTG)29(CTC)589(CTG)29(CG)2(CTG)24	672	340-804	T2T	E2
dm_34	(CTG)91; (CTG)85; (CTG)83	91; 85; 83	82-147	T2T	E1
dm_35	/	/	436-475	T2T	E2
dm_36	/	/	377-515	T2T	E2
dm_37	/	/	185-466	T2T	E2
dm_38	/	/	119-175	T2T	E1
dm_39	/	/	331-889	T2T	E3
dm_40	/	/	414-763	T2T	E2
dm_41	/	/	130-182	T2T	E1
dm_42	/	/	133-229	T2T	E1
dm_43	/	/	133-158	T2T	E1
dm_44	/	/	189-283	T2T	E2
dm_45	/	/	204-506	T2T	/
dm_46	/	/	222-286	T2T	E2
dm_47	/	/	251-587	T2T	E2
dm_48	/	/	218-499	T2T	E2
dm_49	/	/	422-575	T2T	E2
dm_50	/	/	205-432	T2T	E2
dm_51	/	/	159-572	T2T	E2

/ not available.

Supplementary Table 8: Prospect of the cohort with DM2. Repeat expansion patterns characterization for DM2 patients in gene *CNBP*. IGV visual inspection (native ONT), IGV (long PCR), SB estimate, and reference genome are reported. All three samples show expansions, confirming the genetic SB diagnosis. Sample pmm_3 show an expansion with a different repeated motif (CTG). However, it could probably be due to an error in the basecalling process.

ID	Disease (gene)	Expansion pattern	IGV (native)	IGV (long PCR)	Reference
pmm_1	DM2 (*CNBP*)	CCTG	504	/	T2T
pmm_2	DM2 (*CNBP*)	CCTG	/	92-105	T2T
pmm_3	DM2 (*CNBP*)	CTG	/	484	T2T

/ not available.

Supplementary Table 9: Repeat size estimated by different variant callers for DM1 cohort (hg38). Repeat expansion patterns characterization for DM1 patients, aligned to hg38 reference genome. IGV is considered as reference, while NanoExpansion provided the entire expanded pattern. The results from Straglr, Sniffles, CuteSV, NanoRepeat, STRdust, STRkit, and LongTR are also reported.

Patient ID	NanoExpansion	IGV	Sraglr	Sniffles	CuteSV	NanoRepeat	STRdust	STRkit	LongTR
dm_1	(CTG)43(CTC)694(CTG)56	796	920		972 (3)	796	1004 (3)		792
	(CTG)1119	1119		1002 (2)		1119		1118	1118
	(CTG)1005	1002				1002		1004	1004
	(CTG)727	745				725			
dm_2	(CTG)1094	1091	1092	1091	1019	1091	1094 (2)	1094	1094
	(CTG)38(CTC)909	948	958			949			946
	(CTC)857(TG)31(GTG)4(GAG)2(CTG)41	938	933						
	(CTG)50(CTC)428	478	465						
dm_3	(CTG)83	83	84			83	75 (5)	83	83
	(CTG)75	74	74		77 (5)	74		75	75
	(CTG)75	73	78			73		75	75
	(CTG)77	77	76			77		77	77
	(CTG)75	75	75	75 (5)		75		75	75
dm_4	(CTG)1100	1100	1096	1096	1096	1096	/	/	/
dm_5	(CTG)137	137	137			135	137 (3)	137	137
	(CTG)127	127	127	126 (3)	129 (3)	125		127	127
	(CTG)123	123	123			122		123	123
dm_6	(CTG)11(CTC)413(CTG)8(CCG)(CTG)8(CCG)(CTG)6(CCG)3(CTG)6(CCG)2(CTG)8(CCG)(CTG	526	527	526	526	526	524	/	524
dm_7	(CTG)466	465	542	465	541 (2)	465	620 (2)	466	464
	(CTG)620	618		618		618		620	620
dm_8	(CTG)146(CTC)344(CTG)2(CCT)4(CTG)144	640				642	767 (4)	/	640
	(CTG)79(CTC)688(CTG)50	817			691 (2)	817			817
	(CTG)59(CTC)610(CTG)1	670	670	675 (2)					670
	(CTG)1241	1241	1251		1250	1241	1242 (2)	1242	1242
	(CTG)16(CTC)1388(CTG)46	1408	1460	2079			1068 (2)		

**dm_9**	(CTG)899(CTC)1(CTG)441	1341	1351	1341		1341			
	(CTG)105(CTC)1143(CTG)9	1257	1258			1257			
	(CTG)83(CTC)936(CTG)38	1056							1052
**dm_10**	(CTG)115(CCG)41(CTG)53	209	209	209	209	209	209	209	209
	(CTC)1198(CTG)28	1233	1244	1234	1214	1235	1226 (2)		1225
**dm_11**	(CTG)799	797	807	797	777	797		799	798
	(CTG)131	131	132	131	131	132	/	131	131
**dm_12**	(CTG)204	204				204			
**dm_13**	(CTG)315	315	315	/	/	/	/	/	/
	(CTG)383	383	393	383	383	383	1053 (3)	383	383
**dm_14**	(CTG)672	672	682	882 (3)	868 (3)	672		672	672
	(CTG)30(CTC)831(CTG)12	882	892						873
	(CTG)1053	1050	1060			1050		1053	1053
	(CTG)25(CTC)948	976	986		868		973 (2)		973
**dm_15**	(CTG)818	816	817	816 (2)		816		818	818
	(CTG)35(CTC)721(CTG)60	812	822		816 (2)				803
	(CTG)199	199	199		189 (3)	199	199 (3)	199	199
**dm_16**	(CTG)165	165	165	165 (3)		165		165	165
	(CTG)159	159	159			159		159	158
	(CTG)117	115	116			115	99 (8)	117	117
	(CTG)10(CTC)53(CTG)33	98	99	98 (10)		98		99	99
	(CTG)34(CTC)39(CTG)56	127	128			127		129	129
	(CTG)110	109	110		104 (10)	109		110	110
**dm_17**	(CTG)98	95	95			94		98	96
	(CTG)134	114	136			135		134	134
	(CTG)93	91	92			91		96	94
	(CTG)94	94	95			94		94	94
	(CTG)94	94	95			95		94	94
	(CTG)94	94	95			96		94	93
	(CTG)47(CTC)337(CTG)86	470	480		1106	470	1118 (6)		469
	(CTG)41(CTC)477(CTG)30	550	560		551 (4)	552			547
	(CTG)66(CTC)496(CTG)10	574	584	574 (4)		574			571
**dm_18**	(CTG)46(CTC)510(CTG)52	611	621			611			608
	(CTG)60(CTC)710(CTG)91(CTC)163(CTG)73	1098	724			1096			1096
	(CTG)1117	1117	1106			1117			1117
	(CTG)727	727							
	(CTG)71(CTC)648(CTC)5	724							
	(CTG)1404	1400	1410		1400	1400	1428 (3)	1404	1404
	(CTG)1002	1001	1011	1001 (2)		1001		1002	1002
**dm_19**	(CTG)888	887	897	594	539	887		888	888
	(CTG)596	594	604			594		596	596
	(CTG)30(CTC)887(CTG)47	968	978		967 (3)	967			963

	(CTG)77(CTC)924(CTG)93	1094	1104			1094			1094
	(CTG)28(CTC)845(A)38(CTC)67(CTG)38	990	1000				990 (8)	/	990
**dm_20**	(CTG)52(CTC)1223	1275	1283	1275	1132	52			1275
	(CTG)92	92	93		95 (2)	92	/	92	92
**dm_21**	(CTG)99	99	99	99 (2)		99		99	99
	(CTG)42(CTC)716(CTG)40	798	808			798	936 (2)	/	797
**dm_22**	(CTG)27(CTC)902(CTG)4	933	943	933 (2)	865 (2)	27			932
**dm_23**	(CTG)47(CTC)6(CTG)4(CTC)277(CTG)52	386	340	386	386	385	388 (2)	/	386
	(CTC)118(CTG)74	191	192	191	191				191
**dm_24**	(CTG)45(CTC)600(CTG)45	690	700	/	/	/	/	/	/
**dm_25**	(CTG)129	129	/	/	/	/	/	/	/
**dm_26**	(CTG)558	558	568	558	534	567	560 (2)	558	558
	(CTG)61(CTC)152(CTG)297	510	519			520		510	509
	(CTG)35(CTC)253(CTG)36	324	334	324	324	325			324
**dm_27**	(CTG)107(CTC)82(C)167(CTC)149(CTG)147	540		540	540		539 (1)		540
	(CTG)246	246	247						
	(CTG)70(CTC)4(CTG)4(CTC)85(CTG)34	197	197	197	197	195		197	197
	(CTG)107	106	106	106	106	105		107	107
**dm_28**	(CTG)307	307	307	307	307	307	/	307	307
**dm_29**	(CTG)77(CTC)2166(CTG)13	2255	2265	2256	2256		2256 (5)		2255
	(CTG)30(CTC)974(CTG)53	1057	1067			1057	828 (5)		1057
	(CTG)36(CTC)788(CTG)4	828	838	828 (2)	879 (2)	819			828
	(CTG)59(CTC)700(CTG)52	811	821						811
	(CTG)15(CTC)446(CTG)27	488	498	488	487	489			488
**dm_30**	(CTG)151	151	151	151	126	151	109 (3)	151	151
	(CTC)69(CTG)51	120		119		115			119
	(CTG)109	109	109			115		109	109
	(CTG)91	91							
**dm_31**	(CTG)514	513	514	513	513	514	/	/	513
**dm_32**	(CTG)4(AAA)66(TTG)2(CTG)33	147	/	147	147	104	104 (2)	/	/
**dm_33**	(CTG)29(CTC)589(CTG)29(CG)2(CTG)24	672	682	672	672	672	672	/	671
**dm_34**	(CTG)91	91	92			86	91 (3)	91	91
	(CTG)85	85	85	85 (3)	86 (3)	86		85	85
	(CTG)83	83	84			86		83	83

/ not available. (2) number of readsshowingthatexpansion.

Supplementary Table 10: Repeat size estimated by different variant callers for DM1 cohort (T2T). Repeat expansion patterns characterization for DM1 patients, aligned to T2T reference. IGV is considered as reference, while NanoExpansion provided the entire expanded pattern. The results from Straglr, Sniffles, CuteSV and NanoRepeat are also reported.

**Patient ID**	**IGV**	**Sraglr**	**Sniffles**	**CuteSV**	**NanoRepeat**
**dm_28**	307	307	307	307	308
**dm_29**	2265	2265	2265	2266	
	1067	1067			1058
	838	838	838		
	821	821		806	821
	498	498	497		504
**dm_30**	146	151		146	152
	115	109	114 (2)		121
	104			109 (2)	110
**dm_31**	508	514	508	508	515
**dm_32**	142	/	102	102	104
**dm_33**	667	682	666	667	673
**dm_34**	27 (8)	27 (8)	27 (8)	27 (8)	28 (8)
**dm_35**	91	91			92
	85	85			86
	83	83	80 (3)	81 (3)	84

/ not available. (2) number of reads showing that expansion.

Supplementary Table 11: Repeat size estimated by different variant callers for ALS cohort. Repeat expansion patterns characterization for ALS patients. IGV is considered as reference, and the entire expanded pattern has been extracted from it. The results from Straglr, Sniffles, CuteSV, NanoRepeat, STRdust, STRkit, and LongTR are also reported.

Patient ID	NanoExpansion	IGV	Straglr	Sniffles	CuteSV	NanoRepeat	STRdust	STRkit	LongTR
als_1	(CCGGGG)65(GAGGGA)65(CTCGGGCTCGGGC)(GGTCGG)47(CCGGGG)214(AGGCGG)10(CCGGGG)81(GGTCGG)29(CGGGCTCGGGCTC)(GGTCGG)28(CCGGGG)206(GGTCGG)2(CCGGGG)91(GGTCGG)4(CCGGGG)100(CCGGG)(CCGGGG)80(GGTCGG)27(CCGGGG)63(GGTCGG)3(CCGGGG)78(GGTCGG)4(CCGGGG)36(GGTCGG)39(CCGGGG)30(GGTCGG)29(CTCGGGCTCGGG)(CCGGGG)100(GGTCGG)(CCGGGG)77(GGTCGG)3(CCGGGG)191(GGTCGG)2(CCGGGG)299(GGTCGG)2(CCGGGG)28(GGTCGG)51(CCGGGG)56(GGTCGG)18(CCGGGG)310(GAGGGA)89(CCGGGG)203(GGTCGG)54(CCGGGG)98(GAGGGA)170	3084	2921	3084	3084	166 (2)	2921	3377	3087
	(CCGGGG)106(CTCCGG)(CCGGGG)753(GAGGGA)191(CCGGGG)176	1226	/	/	/	/	/	/	/
als_2	(CCGGGG)140(GGTCGG)(CCGGGG)38(GGTCGG)4(CCGGGG)12(CCGGG)(CCGGGG)33(GGTCGG)2(CCGGGG)101(GGGTCGG)4(CCGGGG)153(AG)(CCGGGG)224(GGTCGG)(CCGGGG)164(CGGGCTCGGG)(CCGGGG)100(GGTCGG)3(CCGGGG)40(GGTCGG)4(CCGGGG)14(GAGT)(CCGGGG)14(GGTCGG)(CCGGGG)57(GGTCGG)3(CCGGGG)12(GGAG)(CCGGGG)15(GGTCGG)2(CCGGGG)282	1421	1431	1421	1421	1457	1431	1534	1424
	(CCGGGG)49(TCGGGGTCGG)2(CCGGGG)13(GGTCGG)2(CCGGGG)76(CTCGGGCTCGCTCGGTCTCGGGCTTCGGGCTCGGGCTCGGCTCGGCTCGGTCGCTCGGGCTCGGGTC)(CCGGGG)38(AG)(CCGGGG)64(GGTCGG)4(CCGGGG)47(CTCGGCTCGGG)(CCGGGG)34(TGGGCTCGGG)(CCGGGG)43(GGTCGG)4(CCGGGG)67	470	/	/	/	/	/	/	/
	(CCGGGG)267(GGTCGG)3(CCGGGG)178	445	/	/	/	/	/	/	/
	(CCGGGG)40(GGTCGG)13(CGGGCGGGCTGGGGCG)(CCGGGG)12(GGTCGG)27(CCGGGG)52(GGTCGG)15(CCGGGG)144	312	/	/	/	/	/	/	/
als_3	(CCCCGG)52(TCG)(CCCCGG)7	59	/	/	/	/	/	/	/
als_4	(CCGGGG)34(CCG)3(CCGGGG)303(GGGCGGG)(CCGGGG)58(GGTCGG)2(CCGGGG)15(GGTCGG)3(CCGGGG)322(GGGGGCGG)(CCGGGG)45(CGGCGGCGGCGG)(CCGGGG)109(GGGGGCGGGG)(CCGGGG)13(GGGGGCGGGG)(CCGGGG)20(GGGCGGGG)(CCGGGG)9(GGGGGCGGGG)(CCGGGG)20(GGGGGCGGGG)(CCGGGG)9(GGGGGCGGGG)(CCGGGG)11(GGGGGCGGGG)(CCGGGG)32(GGTCGG)3(CCGGGG)250(GGGGGCGGGG)(CCGGGG)44	1346	1341	1331	1331	1365 (2)	1340	1432	1333
	(CCGGGG)150(GGTCGG)3(CCGGGG)179(GGTCGG)3(CCGGGG)426(GGTCGG)6(CCGGGG)183	948	948	/	/	/	/	/	/
als_5	(CCGGGG)41(GGTCGG)4(TCACCTCA)(CCGGGG)67(GGTCGG)(CCGGGG)100(GGTCGG)4(CCG)5(CCGGGG)82(GGC)(CCGGGG)58(AG)(CCGGGG)6(GGTCGG)6(CCGGGG)72(CAGGTCGG)4(CCGGGG)110(GGGCGGGCA)(CCGGGG)22(GCGGTCGG)3	587	587	/	/	587	/	/	/
als_6	(CCCCGG)589	589	596	/	/	/	/	/	/
	(CCCCGG)120(GGC)(CCCCGG)194(GGG)(CCCCGG)21(GGTCGG)4(CCCCGG)66	407	409	/	/	/	/	/	/
	(CCCCGG)20(AG)(CCCCGG)70(GGTCGG)4(CCCCGG)245	340	339	/	/	/	/	/	/
als_7	(CCCCGG)19(TGGCGGGC)(CCCCGG)9(GGTCGG)4	33	/	/	/	/	/	/	/
als_8	(CCCCGG)18(CGGGGGCGGTG)(TGAG)8	32	/	/	/	/	/	/	/
als_9	(CCGGGG)26	26	21	26	27	26	27	35	26

/ not available.

Supplementary Table 12: Repeat size estimated by different variant callers for test cohort. Repeat expansion patterns characterization for patients of the test cohort (SCA, CANVAS, HD, and Kennedy). IGV is considered as reference, and the entire expanded pattern has been extracted from it. The results from Straglr, Sniffles, CuteSV, NanoRepeat, STRdust, STRkit, and LongTR are also reported.

Patient ID	NanoExpansion	Gene	Disease	IGV	Straglr	Sniffles	CuteSV	NanoRepeat	STRdust	STRkit	LongTR
**sc_2**	(ATTTT)99(ATTCC)111(ATTTT)9	*DAB1*	SCA37	220	218	220	220	/	218	/	/
**sc_3**	(TGGAA)463	*BEAN1*	SCA31	460	463	460	460	/		463	459
	(TGGAA)38(ATTTT)3(TGGAA)410			434	451				451		
**sc_4**	(CAG)72	*ATXN3*	SCA3	69	72			67 (3)	/	72	/
	(CAG)71 (3)			67 (3)	71	67 (4)	67 (4)				
**sc_5**	(GAA)381	*FGF14*	SCA27	375	379	362 (8)	362 (9)	396	369	368	375
	(GAA)377			364	375			380		324	365
	(GAA)374			362	368			372		317	362
	(GAA)366			358	366			368		310	358
	(GAA)364			355	362			362		307	356
	(GAA)360			354	359			360		302	355
	(GAA)357			353	358					300	347
	(GAA)356			350	351			351		294	
	(GAA)348			340	350						
**sc_6**	(GAA)227	*FGF14*	SCA27	226	230	223 (5)	222 (6)	226 (4)	226	230	226
	(GAA)225			225	227	146 (1)				227	224
	(GAA)223			223	224 (3)					224	221
	(GAA)221 (3)			220 (3)	206					224	220
**sc_7**	(GAA)284 (4)	*FGF14*	SCA27	284 (4)	287 (4)	283	284	290 (3)	288	83	285 (3)
								288			292
**sc_8**	(GAA)333	*FGF14*	SCA27	331	327	324	320	336	327	330	/
	(GAA)329			327	327			332		328	
	(GAA)329			327	326			332		328	
	(GAA)328			326	325			331		324	
	(GAA)327			325	321			331		317	
	(GAA)323			323	315			327		298	
	(GAA)323			321	311			326		288	
	(GAA)317			315	302			320			
	(GAA)304			302	301			301			
**sc_9**	(TGGAA)160	*BEAN1*	SCA31	161	163	159	/	/		/	162
	(TGGAA)160			160	161						160
	(TGGAA)159			143	159				147		158
**sc_12**	(ATTTT)125(ATTCC)163(ATTTT)91	*DAB1*	SCA37	380	379	380	380	380	379	/	378
**cnv_1**	(GGGAA)393(GA)(GGGAA)710	*RFC1*	CANVAS	1104	/			/	1156	/	
	(GGGAA)388(AAAGAAA)(GGGAA)717			1109							1033
	(GGGAA)845(GAAAT)(GGGAA)15(CGGGC)18(GGCT)172			1017			1020 (3)				1033
	(GGGAA)399(GGC)15(GGCT)50(GGC)7(GGCT)114(GGGAA)211(AGGC)1			995		990 (1)					

	0(GGCT)185(GGAAA)55(GGAG)(GGGAA)24<br>(GGGAA)10(AAA)(GGGAA)296(GAGCTAAGG)(GGGAA)390			701							
**cnv_2**	(GGGAA)176(GGC)(GGGAA)805<br>(GGGAA)86(GAGGCGAGG)2(GGGAA)391<br>(GGGAA)219(GAAAA)(GGGAA)213	*RFC1*	CANVAS	985<br>486<br>434	983<br>481<br>434	/	/	/	/	/	/
**cnv_3**	(GGGAA)166(GAGGGG)(GGGAA)18(AGCCTA)93(GGGAA)4(AGCCTA)8(GGGAA)80(AGCCTA)102(GGGAA)208(AGCCTA)4(AGCCTAT)64(GGGAA)19(AGCCTA)40(GGGAA)106<br>(GGGAA)884<br>(GGGAA)650	*RFC1*	CANVAS	979<br>873<br>640	/	978 (3)<br>639 (2)	830 (3)	/	921	/	946<br>668
**hd_1**	(CAG)47<br>(CAG)45<br>(CAG)44	*HTT*	HD	47<br>45<br>45	47<br>45 (2)	45 (3)	45 (3)	48<br>47<br>46	47	48<br>46<br>46	47<br>45<br>45
**kn_1**	(CAG)48 (2)<br>(CAG)47	*AR*	KENNEDY	46 (2)<br>45	46 (2)<br>45	72 (3)	/	50	44	/	44 (2)<br>43

/ not available. (2) number of reads showing that expansion.

Supplementary Table 13: Comparison between variant caller tools and IGV results. Metrics computed to compare variant caller tools to IGV, considered the ground truth. Metrics are reported as mean values and 95% confidence intervals. LongTR and NanoExpansion clearly outperformed the other softwares (lower error metrics), approaching what can be visually inspected from IGV.

Software	HR	MAE	RMSE	Bias	Std error	IQR	CV	weighted-MAE	weighted-RMSE	CS	ICC	CCC
**Straglr**	0.801	11 [6, 18]	38 [11, 62]	0.66 [-6.8, 5.9]	38.5	10.0	0.076	13.7	47.9	0.067	0.99 [0.98, 0.99]	0.99 [0.99, 0.99]
**Sniffles2**	0.648	24 [5, 49]	98 [21, 162]	5.9 [-14.3, 33.7]	99.1	0.75	0.17	36.3	152.0	0.026	0.98 [0.95, 0.99]	0.98 [0.95, 0.99]
**CuteSV**	0.648	40 [18, 68]	109 [44, 168]	-0.086 [-26.2, 31.5]	109.7	9.5	0.18	62.04	167.9	0.016	0.98 [0.93, 0.99]	0.98 [0.93, 0.99]
**NanoRepeat**	0.682	49 [3, 116]	313 [7, 518]	-44.6 [-113.4, 1.3]	312.1	2.0	0.62	71.95	460.4	0.014	0.73 [0.43, 0.99]	0.73 [0.43, 0.99]
**STRdust**	0.580	68 [30, 116]	163 [71, 239]	26 [-19, 79]	163.4	8.5	0.24	117.8	282.2	0.008	0.96 [0.89, 0.99]	0.96 [0.88, 0.99]
**STRkit**	0.449	15 [7, 27]	46 [19, 70]	-0.46 [-9.9, 9.8]	46.4	2.0	0.12	34.4	102.7	0.027	0.99 [0.99, 0.99]	0.99 [0.99, 0.99]
**LongTR**	0.676	2.9 [1.7, 4.8]	9.0 [3.7, 13.8]	-0.62 [-2.4, 0.79]	8.99	2.0	0.017	4.4	13.3	0.17	0.99 [0.99, 0.99]	0.99 [0.99, 0.99]
**NanoExpansion**	0.983	3.5 [2.4, 4.8]	8.8 [5.5, 12.0]	-0.09 [-1.5, 1.2]	8.85	2.0	0.017	3.6	8.9	0.22	0.99 [0.99, 0.99]	0.99 [0.99, 0.99]

[,] 95% confidence interval.

Supplementary Table 14: Short and long read sequencing comparison. Comparison between short and long read sequencing (ONT) for each locus investigated in five cases inlcuding healthy controls. Some alleles were unavailable (NA) for short read sequencing due to intrinsic limitation derived from 150 cycles in Illumina exome sequencing protocol (ILM).

chromosome	gene	disease	location/direction	precise coordinate	Normal range	Pathological	Healthydonor1 ILM	Healthydonor1 ONT	sc_5 exome	sc_5 ONT	sc_12 exome	sc_12 ONT	sc_8 exome	sc_8 ONT	sc_13 exome	sc_13 ONT
chr19:45,769,717-45,782,490	*DMPK*	DM1	3'UTR/-	chr19:45770200	5-37	>50-10000	5;26	5;26	12	5;12	NV	5	5;13	5;13	5;6	5;6
chr3:129,167,827-129,183,896	*CNBP*	DM2	intron/-	chr3:129172650	11-30	>50	NV	15;20	NV	23;24	NV	29;31	25	24;25	NV	25;27
chrX:67,544,021-67,730,619	*AR*	Kennedy disease	coding/+	chrX:67545320	9-36	>38-68	22	22	21	21	NV	16;25	22;24	22;24	23;24	23;24
chr9:27,546,546-27,573,481	*c9orf72*	ALS	5'UTR intron/-	chr9:27573523	3-25	>25	NV	4;5	NV	1;3	5;10	5;10	NV	2;6	8	6;8
chr6:16,299,112-16,761,460	*ATXN1*	SCA1	coding/-	chr6:16327700	6-38	>39-88	27;29	27;29	29;30	29;30	26;28	26;28	29;30	29;30	29;30	29;30
chr12:111,452,214-111,599,315	*ATXN2*	SCA2	coding/-	chr12:111599000	13-31	>32-500	21;22	21;22	21;22	21;22	22;23	22;23	21;22	21;22	15;24	15;24
chr14:92,058,552-92,106,582	*ATXN3*	SCA3	coding/-	chr14:92071010	12-44	>55-887	22;23	22;23	11;12	11;12	7	7;23	22;23	22;23	23	23;28
chr19:13,206,442-13,506,479	*CACNA1A*	SCA6	coding/-	chr19:13207883	4-18	>20-33	11;13	11;13	10;11	10;11	11	11	7;11	7;11	7;11	7;11
chr3:63,863,867-64,003,462	*ATXN7*	SCA7	coding/+	chr3:63912707	4-33	>37-460	9;10	9;10	9;10	10	10;12	10;12	9;10	9;10	9;10	9;10
chr6:170,554,369-170,572,859	*TBP*	SCA17	coding/+	chr6:170561947	25-40	>43-66	35;37	35;37	36;37	36;37	36;38	36;38	36;37	36;37	35;39	35;39
chr20:2,652,632-2,658,393	*NOP56*	SCA36	intron/+	chr20:2652740	5-14	>650-2500	4	4	4	4;8	4;7	4;7	4;6	4;6	5;6	5;6
chr8:104,489,236-104,589,258	*LRP12*	ALS2	5'UTR/-	chr8:104588983	13-45	>90-130	15	15	14;15	14;15	9;12	9;12	9;13	9;13	16	16;17
chrX:147,911,919-147,951,125	*FMR1*	FRAXA	5'UTR/+	chrX:147912082	5-50	>200	20	20	NV	NV	19	NV	NV	30;31	16	16
chr4:39,287,456-39,366,362	*RFC1*	CANVAS	intron/-	chr4:39,348,420	variable	>400AAGGG	NV	12	NV	108;513	NV	11;691	20	20;126	NV	11;108
chr9:69,035,752-69,079,076	*FXN*	Friedrich's ataxia	intron/+	chr9:69037285	5-34	>66-1300	NV	10;20	NV	16;17	NV	9;21	NV	9;10	NV	9;20
chr13:101,708,804-102,404,443	*FGF14*	SCA27	intron/-	chr13:102161575	5-250	>250	NV	11 (1)	NV	10;360	NV	16;17	NV	9;327	NV	44;290

chr1:56,994,778-57,424,060	*DAB1*	SCA37	intron/-	chr1:57367024	7-400(ATTTT)	ATTTC>31-75	NV	14	NV	15;16	NV	79;380	NV	16;99	NV	16;24
chr16:66,427,295-66,482,833	*BEAN1*	SCA31	intron/+	chr16:66490397	variable	TGGAA>110(+TAGAA)	NV	13	NV	13;25	NV	15;133	NV	17;18	15	15;25
chr22:45,671,834-45,845,307	*ATXN10*	SCA10	intron/+	chr22:45819146	10-32	>280	NV	NV	NV	10;12	NV	11;17	NV	9;14	NV	11;14
chr5:146,580,742-146,878,757	*PPP2R2B*	SCA12	5'UTR/-	chr5:146878727	4-32	>43	10;11	10;11	9;10	9;10	10;15	10;15	9;15	9;15	10;15	10;15
chr13:70,099,727-70,157,682	*ATXN8/ ATXN8OS*	SCA8	3'UTR/+	chr13:70139400	15-50	>74-250	23;25	23;25	27	24;27	NV	25;100	23	18;23	24;26	24;26
chr4:3,074,681-3,243,957	*HTT*	HTT	coding/+	chr4:3074904	6-35	>36-250	19;24	19;24	17;19	17;19	19;20	19;20	19;22	19;22	18;19	18;19
chr12:6922459-6944321	*ATN1*	DRPLA	coding/+	chr12:6936750	3-35	48-93	12;14	12 (1)	14;18	14;18	21;24	21;24	18;20	18;20	20;22	18
chr16:72,782,885-73,048,128	*ZFHX3*	SCA4	coding/+	chr16:72787695	14-26	>42-74	21	21	20;21	20:21	21	10;21	20;21	21	17;21	17
chr21:43,773,950-43,776,308	*CSTB*	Unverricht-Lundborg (ULD)	5'UTR/-	chr21:43776462	2-3	>30	NV	NV	NV	NV	2	2	2;3	2;3	2	2
chr16:67,842,320-67,844,195	*THAP11*	SCA	coding/-	chr16:67842894	20-38	>45-100	NV	28;29	29	29	NV	28;29	27;28	27;28	28;29	29

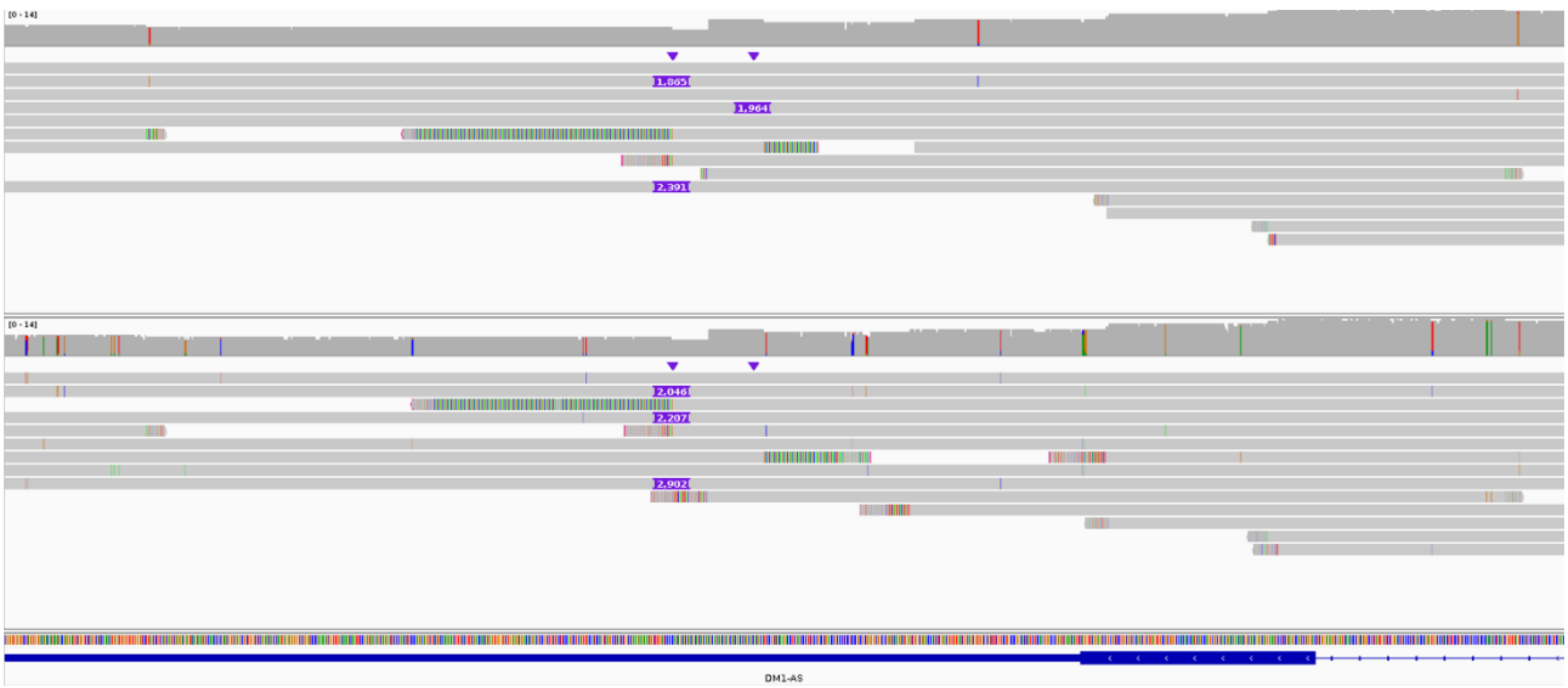

Supplementary Figure 1: Dorado sup model vs dorado hac model. IGV screenshot of a DM1 patient basecalled and aligned using sup (top) and hac (bottom) models. Lots of false positive SNPs disappear using sup basecalling, indicating that sup model improved the overall alignment to reference genome.

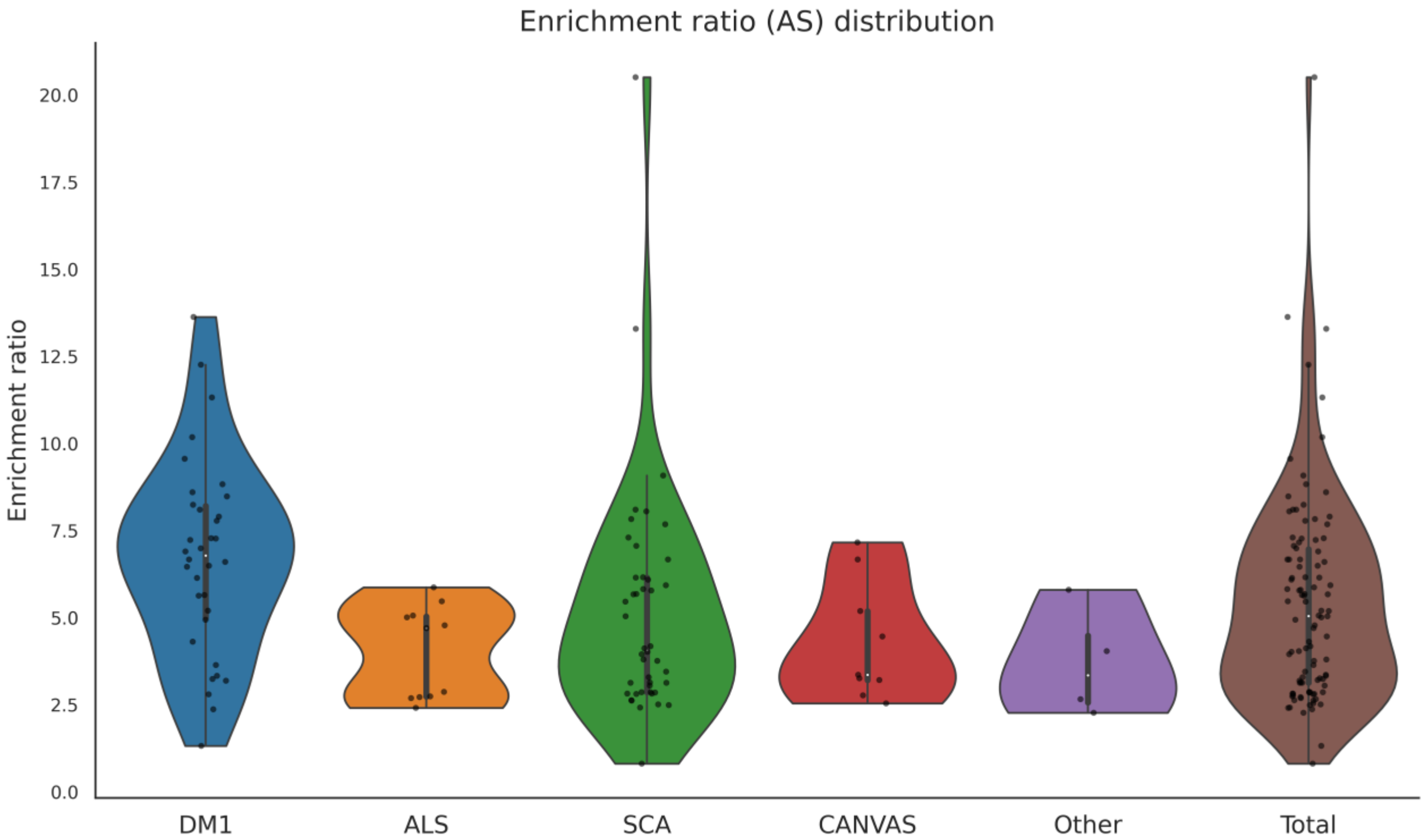

Supplementary Figure 2: Enrichment ratio (AS) distribution. Violin plots of the distribution of the enrichment ratio due to AS (hg38 and T2T) for each disease group. The use of AS clearly enriched for the targets of interest.

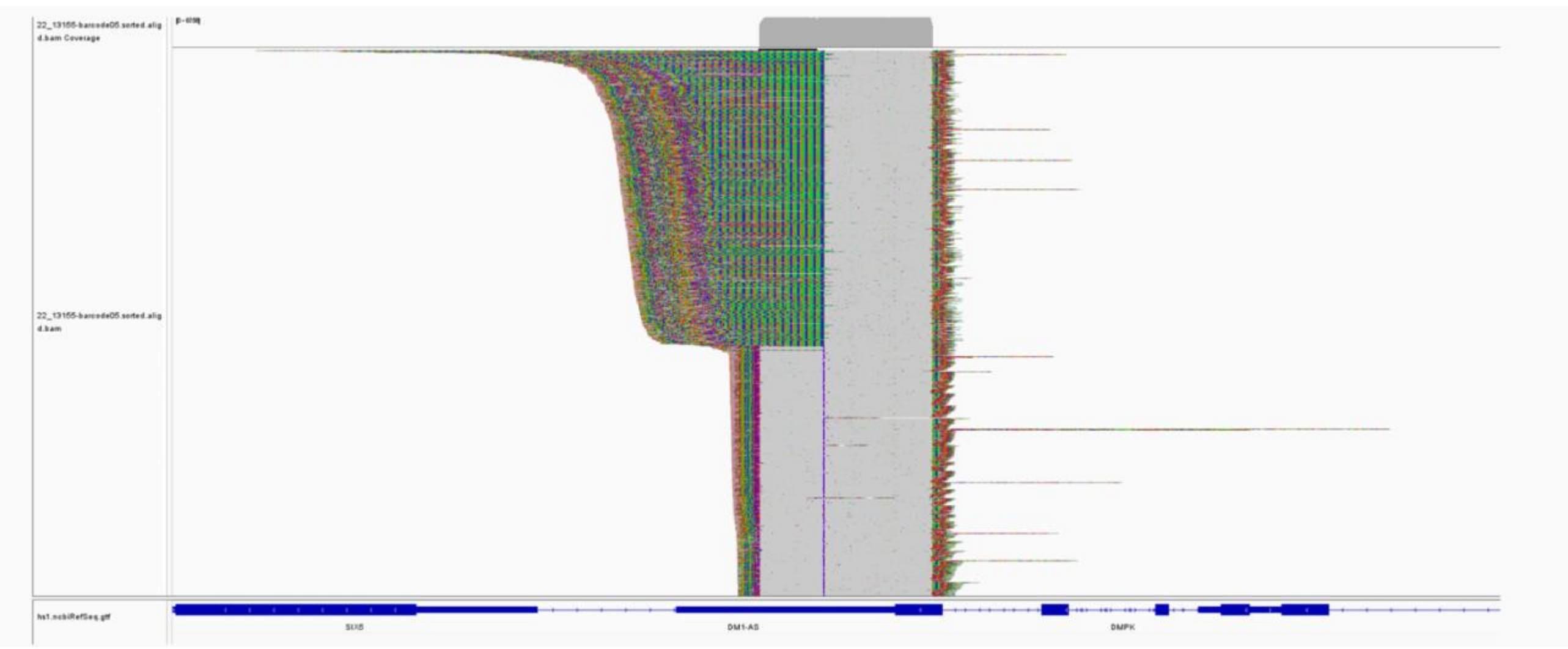

Supplementary Figure 3: Nanopore sequencing with long PCR (DM1). IGV screenshot of a sample with DM1 sequenced using nanopore longPCR and aligned to T2T reference genome. The repeat expansions are clearly visible. As expected, reads vary in size, due to partial polymerization of the region of interest by polymerase enzyme during the PCR process. This made the estimation size more complex and only reads covering flanking regions and including primers were considered.

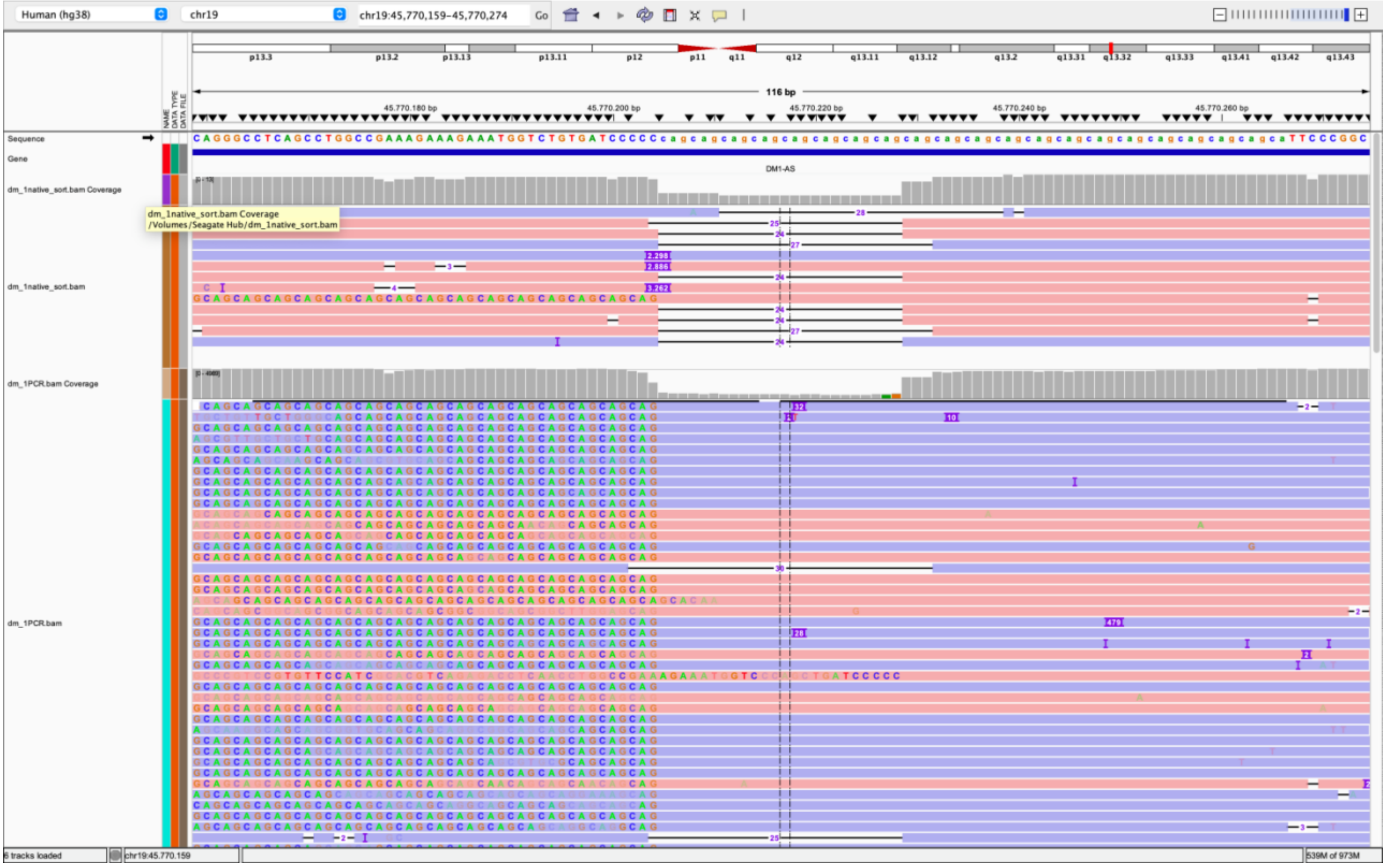

Supplementary Figure 4: Native vs long PCR nanopore sequencing. Comparison between native DNA (top) and long PCR (bottom) analysis of case dm_1 affected by DM1 visualized on IGV. The wild type allele had 12 repeats, while the mutant allele showed large expansions with more than 920 CTG with interruption patterns (see Table S7).

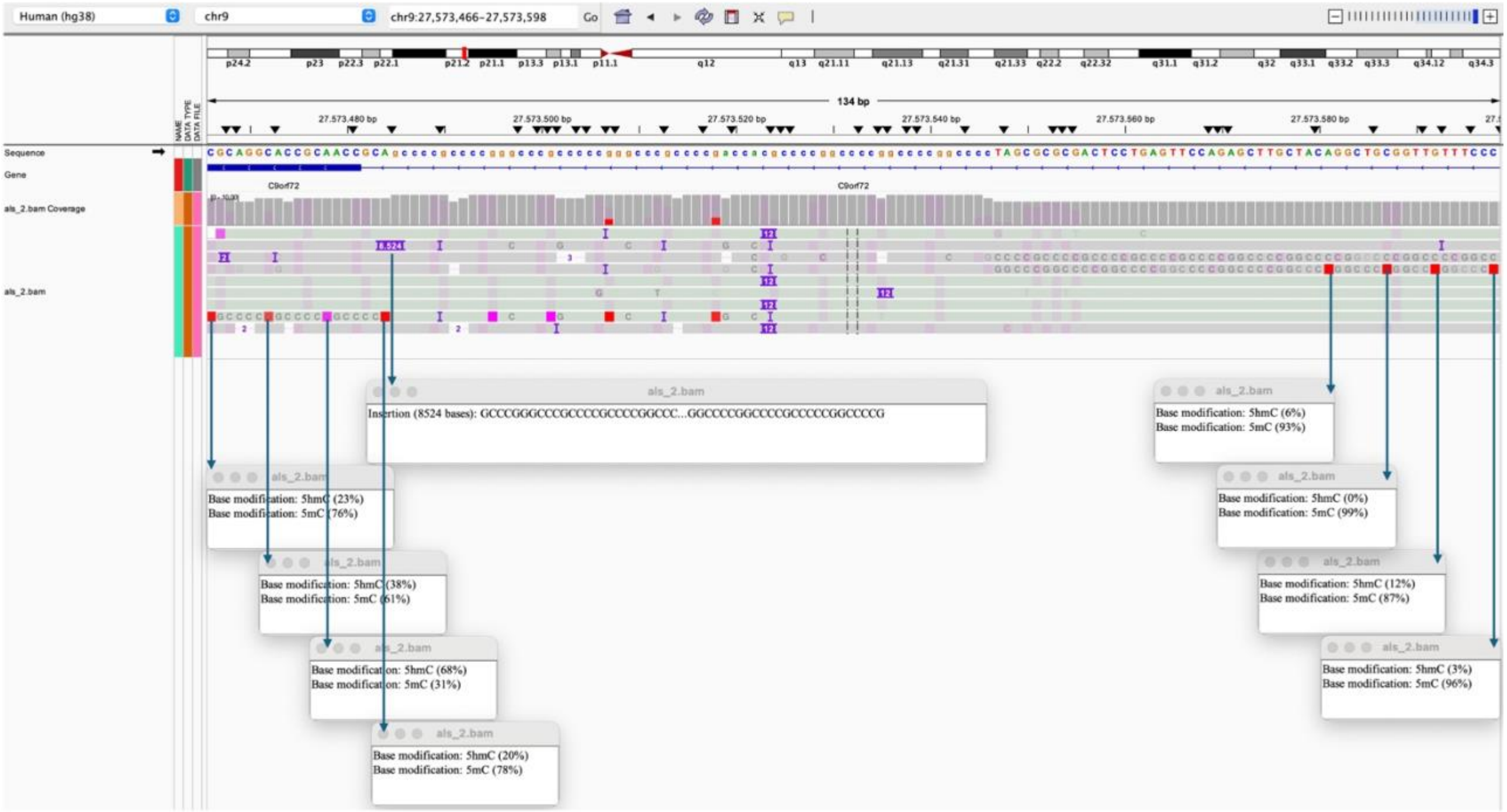

Supplementary Figure 5: Expansion at*C9orf72* for ALS sample.*C9orf72*expansion for case als_2: an insertion of 8524 bp is shown with 1421 CCCCGG repeats; three reads were also expanded, while five reads had an insertion of 12 bp that refers to the wild type allele with a total of five repeats; red squares indicate the prevalence of 5mC, while pink squares the prevalence of 5hmC in percentage as detected by dorado using hg38 as a reference.

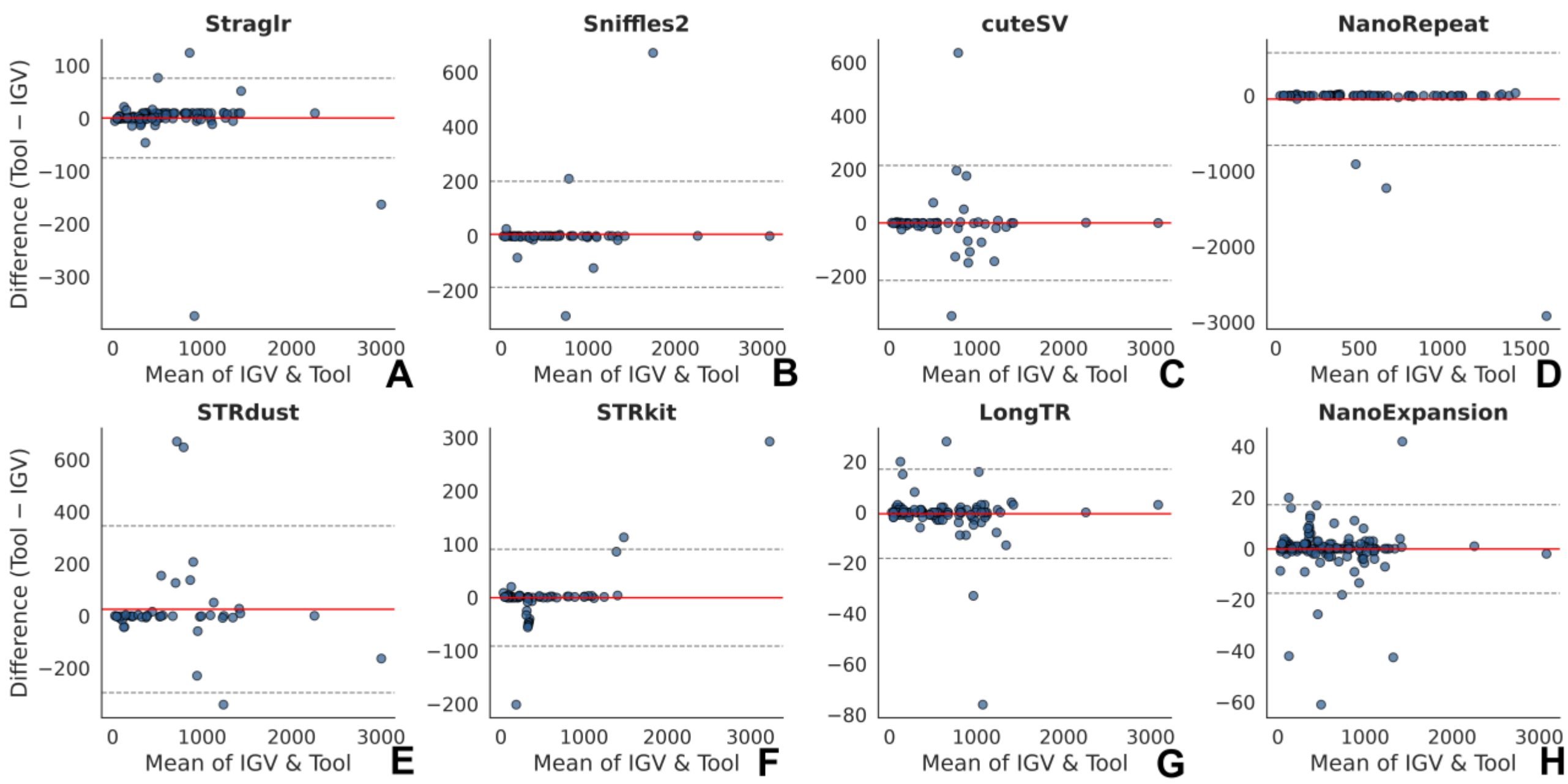

Supplementary Figure 6: Performances of variant caller software tools. Bland-Altman plots of the comparison between IGV estimates of expansion size (taken as ground truth) and estimates obtained through **(A)** Straglr, **(B) Sniffles2**,**(C)** cuteSV, (**D**) NanoRepeat, (**E**) STRdust, (**F**) STRkit, (**G**) LongTR, and (**H**) NanoExpansion. With respect to LongTR and NanoExpansion, the other softwares underperformed, being characterized by higher bias (see Table S13).
